# Digital Immunoassay for Biomarker Detection Based on Single-Particle Laser Ablation ICP MS

**DOI:** 10.1021/acs.analchem.5c00641

**Published:** 2025-06-25

**Authors:** Vilém Svojanovský, Jakub Máčala, Antonín Hlaváček, Aleš Čermák, Jaromír Stráník, Pavel Bouchal, Ivana Mašlaňová, Petr Skládal, Zdeněk Farka, Jan Preisler

**Affiliations:** 1 Department of Chemistry, Faculty of Science, 37748 Masaryk University, Kamenice 5, Brno 625 00, Czech Republic; 2 Department of Biochemistry, Faculty of Science, 37748 Masaryk University, Kamenice 5, Brno 625 00, Czech Republic; 3 Institute of Analytical Chemistry of the Czech Academy of Sciences, Veveří 97, Brno 602 00, Czech Republic; 4 Urology Clinic, 48243 University Hospital Brno, Jihlavská 20, Brno 625 00, Czech Republic; 5 Department of Experimental Biology, Faculty of Science, 37748 Masaryk University, Kamenice 5, Brno 625 00, Czech Republic

## Abstract

Single-particle (digital) immunoassays offer significantly lower limits of detection (LODs) than traditional immunoassays, making them suitable for the detection of low-abundance biomarkers. The most common approach for digital detection is based on counting individual labels. Here, we introduce a novel dot-blot particle-linked immunosorbent assay (PLISA) with digital readout utilizing laser ablation (LA) of photon upconversion nanoparticle (UCNP) labels from the nitrocellulose substrate. Compared to conventional LA, our approach allows desorption of intact nanoparticles and their precise counting by single-particle inductively coupled plasma mass spectrometry (SP ICP MS), thus counting individual UCNP-labeled immunocomplexes. Digital signal processing filters instrument noise and nanoparticle aggregates, minimizing potential errors. The immunoassay and LA SP ICP MS readout were optimized using human serum albumin, a kidney damage biomarker, as a model analyte, obtaining LODs of 0.18 and 0.12 ng/mL for the reference upconversion luminescence (UCL) and LA SP ICP MS readout, respectively. Building upon these optimized conditions, we developed PLISA for prostate-specific antigen, the key prostate cancer biomarker, with LODs of 2.4, 1.4, and 0.3 pg/mL for the UCL, analog, and digital LA SP ICP MS readout, respectively. The LOD in the sub-pg/mL range highlighted the advantage of particle counting and its ability to detect low-abundance biomarkers, as superior performance was achieved compared to the UCL and analog LA ICP MS readout. Finally, clinical serum samples of patients tested for prostate cancer were analyzed, and a strong correlation with the reference electrochemiluminescence method confirmed the potential of LA SP ICP MS for clinical diagnostics.

## Graphical abstract

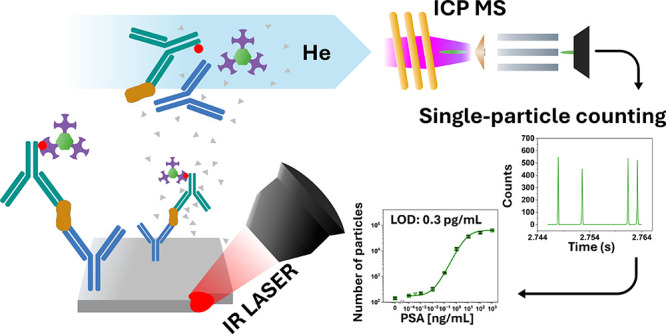

## Introduction

Immunoassays are routinely used bioanalytical techniques, combining antibodies as biorecognition elements capable of specific binding to target antigens with various labels for signal generation.^1^ Heterogeneous immunoassays, representing the most common format, are performed on the surface of a solid phase, which allows for washing out of the unbound reagents, leading to a decrease of the background signals caused by nonspecific binding. Consequently, background reduction allows for achieving lower LODs.^2^

One of the heterogeneous immunoassay types is dot-blot, representing a simple assay format performed on the membrane surface, where the capture immunoreagents are adsorbed by noncovalent interactions.^3^ Dot-blot immunoassays were successfully used to detect numerous analytes, including biomarkers,^4^ infectious agents,^5^ and organic pollutants;^6^ however, the detection is mostly only semiquantitative.^7^

Several types of labels have been frequently used in dot-blot immunoassays. Enzymes^8^ and fluorophores^9^ are commonly employed for signal generation due to their effectiveness; however, they come with certain drawbacks, such as limited stability of enzymes and photobleaching of molecular fluorophores. To address these drawbacks, various nanoparticle (NP)-based labels have emerged in recent decades, offering enhanced stability and signal intensity, with some even enabling the quantitative analyte detection in dot-blots.^4,10^ For example, gold NPs (AuNPs) were used as colorimetric labels,^11^ and quantum dots as a replacement for organic fluorophores.^12^ Photon upconversion nanoparticles (UCNP) represent another class of nanomaterials recently used as a label in dot-blot immunoassays.^4^ UCNPs are nanocrystals composed of inorganic matrix (e.g., NaYF_4_) doped with lanthanide ions (e.g., Yb^3+^, Er^3+^, Tm^3+^). Their main advantage is connected with the anti-Stokes emission of light of shorter wavelength under the excitation with light of longer wavelength, usually infrared.^13^ Therefore, using UCNP labels reduces background signal, as fluorophores naturally present in biological samples are not excited by infrared radiation.^14^ Thus, immunoassays utilizing UCNP labels can achieve lower LODs, making them suitable for ultrasensitive quantitative analysis of various biomarkers.^15^ Using optical microscopy, even individual UCNPs can be detected, enabling the development of single-molecule immunoassays.^14^

Detection of various types of NP labels can be combined with different readout methods, e.g., absorbance and fluorescence or surface-enhanced Raman spectroscopy.^16^ Another option for the sensitive detection of various NP-based labels containing suitable metals, is laser ablation inductively coupled plasma mass spectrometry (LA ICP MS). Simple chelate labels, which carry only a single metal atom, are commonly used but provide limited sensitivity.^17^ To amplify the signal, polymer chelates like Maxpar, binding around 30 metal atoms, have been developed.^18^ However, the most effective signal amplification comes from metal NPs, which offer the highest number of metal atoms per label size.^19^ Although the incorporation of metal NP resulted in enhanced signals, conventional LA lacked the capability of individual label detection.

Nanomaterials of various compositions and shapes can be analyzed by a single-particle ICP MS (SP ICP MS). This extension of the established ICP MS technique enables the detection of individual NPs, determining their elemental composition, concentration, and size if the shape is known.^20^ After introducing an NP into the plasma, it is atomized and ionized to form an ion cloud, which is recorded as a distinct peak upon reaching the detector.^21^ In the overwhelming majority of cases, NPs are introduced into the plasma by nebulization from a dispersion.^22,23^ The use of laser ablation (LA) for introducing intact NPs was reported in 2016; AuNPs (56 and 86 nm in diameter) were introduced directly into the ICP MS from the polyethylene terephthalate glycol that mediated the desorption and prevented NP disintegration, achieving a detection efficiency of 61%.^24^ Maintaining the UV laser fluence below 0.15 J/cm^2^ was crucial to ensure the desorption of intact AuNPs, as commonly used fluences exceeding 1 J·cm^–2^ would lead to NP disintegration during ablation. In 2022, utilizing an IR laser (2940 nm) allowed the efficient release of intact NPs from biological tissue due to the large difference in absorption of the tissue and NPs. More than 80% of 20 nm AuNPs were detected from labeled cancerous biological tissue, enabling digital mapping of biomarkers in colorectal carcinoma cells.^25^

The application of SP ICP MS in immunoassays holds significant potential, as it allows counting individual biomolecules by utilizing NP-based labels. So far, SP ICP MS has only been employed in immunoassays performed in the liquid phase. Magnetic particles combined with ZnSe quantum dots were used in SP ICP MS to detect carcinoembryonic antigen (CEA).^26^ For the immunoanalysis of rabbit IgG, AuNPs were used as labels in sandwich immunoassays on microtiter plates (MTP). Immunocomplexes were released from MTP through sonication and subsequently analyzed by nebulizer SP ICP MS.^27^ However, the direct introduction of intact NP labels from the solid phase has not been reported yet, even though ablation often leads to higher transport efficiency of the material to the ICP MS and eliminates the need for additional sample handling. The disintegration of NPs during the ablation is a significant challenge, particularly when utilizing conventional LA systems.^25^ An alternative approach, laser desorption/ionization mass spectrometry (LDI MS), has been shown to detect individual NPs. However, LDI MS does not provide the precise counting of individual NPs per pixel as LA SP ICP MS, limiting its use in analyses that require an exact NP counting.^28,29^

In this work, we introduce a novel dot-blot, particle-linked immunosorbent assay (PLISA) method utilizing LA SP ICP MS. The single-particle readout allows the detection of single immunocomplexes labeled with UCNP-streptavidin conjugates (UCNP-SA). Individual UCNPs are introduced into the plasma torch and counted (Figure 1). The potential of PLISA was tested by the detection of human serum albumin (HSA), a biomarker of kidney damage when found in urine, and prostate-specific antigen (PSA), a key serum biomarker of prostate cancer. The performance of UCNP counting was compared to conventional analog signal recording of ICP MS and upconversion luminescence (UCL) scanning as a reference method, demonstrating the potential of LA SP ICP MS for ultrasensitive analysis. Finally, the clinical applicability of PLISA was confirmed by the detection of PSA in serum samples of patients tested for prostate cancer.

**Figure 1:**
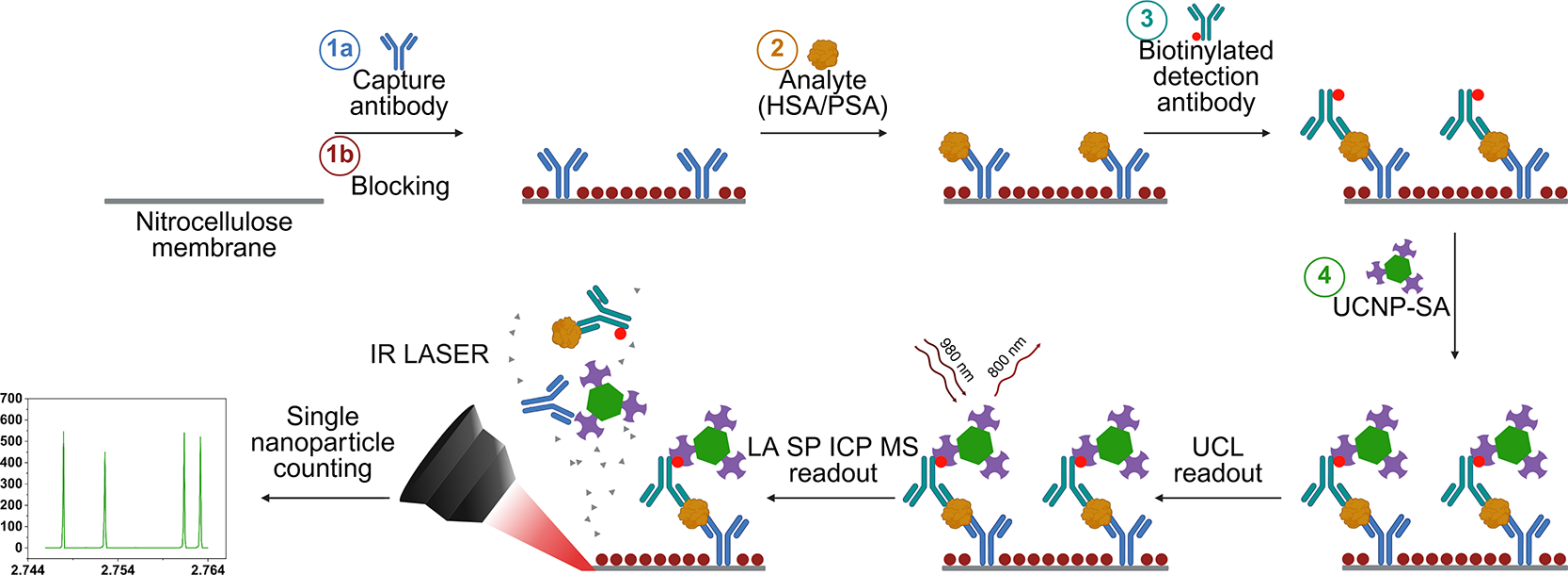
Scheme of the dot-blot immunoassay procedure: (1a) coating of nitrocellulose pads with a capture antibody, (1b) blocking of the surface, (2) incubation with the analyte (HSA or PSA), (3) incubation with biotinylated detection antibody, (4) incubation with UCNP-SA labels. A readout was first performed using UCL scanning and subsequently using LA SP ICP MS. (Created with BioRender)

## Experimental Section

### Immunoassay Preparation

Protocols for the synthesis of NaYF_4_:Yb^3+^,Tm^3+^-based UCNPs (diameter of 64 nm) and their conjugation with streptavidin via an alkyne-PEG-neridronate linker, characterization of the UCNPs and UCNP-SA conjugate using transmission electron microscopy (TEM), dynamic light scattering (DLS) and zeta potential analysis, biotinylation of anti-HSA detection antibody and details on chemicals and materials are provided in the Supporting Information (SI).

All immunoassay steps were done at room temperature unless noted otherwise. The capture monoclonal antibody (AL-01 or ab403 for HSA or PSA detection, respectively) was diluted with the coating buffer to a concentration of 0.5 mg/mL. Subsequently, the antibody solution was dispensed onto nitrocellulose pads of the glass slide by the piezo-driven dispensing system sciFLEXARRAYER S3 (Scienion, Germany), utilizing the piezo dispense capillary PDC70 (Scienion, Germany), producing ∼ 350 pL droplets. One assay spot consisted of 100 droplets, dispensed in 4 consecutive series of 25 droplets with a 10-s delay between each dispensing. After the spotting, the pads were left to dry for 30 min. Then, a gasket was assembled to separate the pads into 64 individual wells, and 65 μL of blocking buffer were added into each well, followed by shaking for 60 min on the orbital shaker at 300 rpm. Then, the blocking buffer was poured out, and the pads were incubated for 30 min with serial analyte dilutions (10^–3^ to 10^5^ ng/mL in the assay buffer for HSA, 10^–4^ to 10^3^ ng/mL in the 50% bovine serum in assay buffer for PSA; 50 μL per well). For the analysis of clinical PSA samples, the tested serum was diluted 100× in 50% bovine serum in assay buffer; this medium introduces a standard matrix and reduces the effect of unknown patient serum dilution. After the incubation, the pads were washed with 65 μL of washing buffer, twice for 1 min, once for 10 min, and three times for 5 min (the washing buffer was exchanged between the individual washing steps), with agitation on an orbital shaker at 300 rpm. Then, the pads were incubated for 30 min with a biotinylated detection antibody (A0433 or BAF1344 for HSA or PSA detection, respectively; 0.5 μg/mL, 50 μL/well), followed by the previously described washing procedure. Afterward, the pads were incubated for 30 min with the UCNP-SA conjugate (6.5 μg/mL, 50 μL/well). Then, the pads were washed using the above-mentioned procedure, with 2 additional 5 min washing steps. After the last washing, the gasket was disassembled, and the slide was dried for 30 min at 40 °C. Subsequently, the UCL (details provided in the SI) and LA SP ICP MS readouts were performed.

### LA SP ICP MS

The samples were ablated using an IR laser ablation system equipped with an optical parametric oscillator (Opolette 2940, OPOTEK, USA) with an output wavelength of 2940 nm, a maximum laser pulse energy of 6 mJ, a pulse width of 5–7 ns, and the output beam diameter of 5 mm. The laser beam was focused to a spot size of 22 μm on the sample, delivering a fluence of 11.4 J/cm^2^ at a repetition rate of 20 Hz. A 75 mm × 25 mm × 1 mm (L × W × H) glass slide containing the sample was inserted into a custom-built aluminum ablation cell equipped with a fused silica slide window for transmission of the laser beam. The ablation lines were spaced 15 μm apart with a travel speed of 150 μm/s for standard LA SP ICP MS scans. Thus, sufficient overlap between the laser pulses was achieved along both the *x*- and the *y*-axis, resulting in a complete ablation of all material from the slide. The pixel size of 15 μm × 15 μm was defined by the travel speed, repetition rate, line spacing, and counting interval. For single-line scans, ablation lines were set 665 μm apart with a travel speed of 15 μm/s. This setup allowed analysis with acceptable particle coincidence and minimal surpassing of peak capacity in the SP mode. The UCNP signals were measured using a quadrupole mass spectrometer (ICP MS 7900, Agilent, USA) at *m*/*z* of 89 (yttrium), with a detector frequency of 10 kHz. Further details of the LA system, its optical parts, and ICP MS are provided in the SI.

### Data Evaluation

ICP MS data were processed using the Analyzer software described in our previous work.^25^ For the LA of UCNPs, as well as the nebulization of UCNPs in the digital mode, i.e., LA SP ICP MS, the signal thresholds were set at 55 and 15 counts, respectively. A signal was classified as a single UCNP if it exceeded the threshold and had a peak width ranging from 3 to 7 data points. Aggregates containing more than 8 UCNPs, i.e., peaks with an area eight times larger than the histogram mode, were not counted. The threshold of 8 was chosen as a compromise based on visual inspection of images and peak area histograms for the whole examined analyte concentration range. A single threshold was used for the whole concentration range to simplify the data evaluation. At low analyte concentrations, it provides a conservative threshold for excluding large aggregates while maintaining an adequate threshold at higher analyte concentrations despite more probable UCNP coincidence. In the analog ICP MS, all data points were accounted for, i.e., the signal was integrated over the specified area. The reconstructed LA SP ICP MS maps were generated in a pseudocolor scale involving ten colors to enhance the contrast; each color corresponds to a specific number of UCNPs per pixel. The selection of area from LA SP ICP MS readout depended on the type of sample preparation. For samples prepared using the piezo-driven dispensing system, quantitation was restricted to UCNPs located within a defined detection area, excluding UCNPs outside this region. In other LA SP ICP MS analyses, all UCNPs detected within the scanned area were counted. Statistical analysis for both the UCL and ICP MS data is described in the SI, see formulas (S1, S2).

## Results and Discussion

### Optimization of LA SP ICP MS

Before starting the assay development, the LA SP ICP MS parameters had to be optimized. During ablation, the sample material is released into the carrier gas. However, not all material is desorbed, transported into the plasma, and detected. Losses may include deposition in the ablation cell or the tubing system and may be affected by the ablation conditions, e.g., He flow rates and the cell geometry. The transport efficiency of the cell in the standard configuration used in this work was 83%, the same value as in the case of the cell with the fast washout configuration.^25^ Details on experiments determining the transport efficiency with statistical evaluation (**S3**) are provided in the SI.

To utilize UCNP labels in immunoassays with LA SP ICP MS readout, the selected dot-blot substrate must meet two critical conditions. First, the substrate must be suitable for the immobilization of proteins, particularly antibodies, and subsequently for the whole immunoassay procedure (e.g., showing low nonspecific adsorption). Second, the substrate must exhibit strong absorption of the laser radiation, which is essential for efficient ablation and subsequent desorption of UCNPs into the carrier gas flow. At the same time, the laser radiation absorbed by the UCNPs should be minimal to prevent their disintegration. UCNPs (NaYF_4_:Yb^3+^,Tm^3+^; characterization by transmission emission microscopy and dynamic light scattering provided in Figure S1) were chosen for this work as they can be detected by both the ICP MS at *m*/*z* of 89 due to their Y content and by UCL due to their luminescence properties. The UCNPs were diluted to 65 ng/L in phosphate-buffered saline (PBS) and characterized by nebulizer SP ICP MS. Their distribution, and low aggregation state are apparent from the histogram of the Y^+^ signal in Figure S2A.

The initial tests were performed with the most frequently used solid phase in heterogeneous immunoassays, polystyrene. UCNPs were diluted in PBS to 2.3 μg/L and dropped onto the polystyrene slide contactless utilizing a syringe (Hamilton, USA) in a volume of 200 nL. Dried droplets were subjected to LA SP ICP MS analysis at a fluence of 5.0 to 11.4 J/cm^2^. However, no signals were detected under these conditions. No ablation occurred at the low fluence levels, while at the high fluence, the polystyrene melted without any noticeable ablation. Even adding 10 mg/mL of glucose absorbing at 2940 nm into the UCNP dispersion did not improve signal detection or the ablation process.

Thus, finding alternative material meeting the criteria for the LA SP ICP MS readout was necessary. Another commonly used solid phase for heterogeneous immunoassays is nitrocellulose. Due to its porous structure, nitrocellulose has the advantage of a larger number of binding sites for the immobilization of biomolecules.^30^ Hence, nitrocellulose pads were tested to assess their suitability as a substrate for ablation and UCNP desorption. Nitrocellulose ablation was found to be insufficient at fluences below 11.4 J/cm^2^. Operating the laser at a fluence of 11.4 J/cm^2^ resulted in the complete removal of the nitrocellulose film and the ablation of intact UCNPs, as evident from the absence of increased background around the peaks of individual UCNPs (Figure S2B). Furthermore, the introduction of UCNPs, as well as AuNPs, through LA enhances the signal compared to a nebulizer (Figure S2). This enhancement is primarily attributed to differences in the ionization mechanisms between dry and wet aerosol, gas composition, and total gas flow. Further discussion is provided in the SI.

### Optimization of PLISA for HSA Determination

The LA SP ICP MS results of dried UCNP droplet samples demonstrated the feasibility of SP detection from the nitrocellulose pads, suggesting the potential of LA SP ICP MS for single-label detection in immunoassays. To test the performance of the method for the detection of individual biomarkers, the UCNP-based dot-blot immunoassay for HSA was performed on nitrocellulose pads. As shown in our previous reports, UCNP-SA conjugates represent suitable labels for the sensitive dot-blot immunoassays with UCL readout.^4,14,31^ However, compared to our previously published dot-blot immunoassay,^4^ additional optimizations were necessary because of the different nitrocellulose membrane type used in this work. The most important was the optimization of the coating of the nitrocellulose pads with the capture antibody, as it is crucial for the successful capture of the target antigen and included volume, concentration and composition of coating buffer as well as concentration of UCNP-SA conjugate and scanning method of LA SP ICP MS (Figures S3–S7). The optimal procedure utilized a piezo-driven dispenser (Figure S8) (details on optimization experiments are provided in SI). This approach resulted in greater consistency and homogeneity of the detection area (Figure 2), leading to an LOD of 0.18 ng/mL for UCL readout and an improved LOD of 0.12 ng/mL utilizing the LA SP ICP MS. The working ranges of this immunoassay were 30–750 ng/mL and 50–3400 ng/mL for UCL and LA SP ICP MS, respectively, indicating the higher accuracy of LA SP ICP MS for higher analyte concentrations. Although the antibody deposition with a pipette on a larger area resulted in a lower LOD for the UCL readout compared to the piezo-driven spotting, incorporating the automatic deposition system significantly improved the consistency of results obtained from both detection methods; therefore, it was used for further experiments.

**Figure 2:**
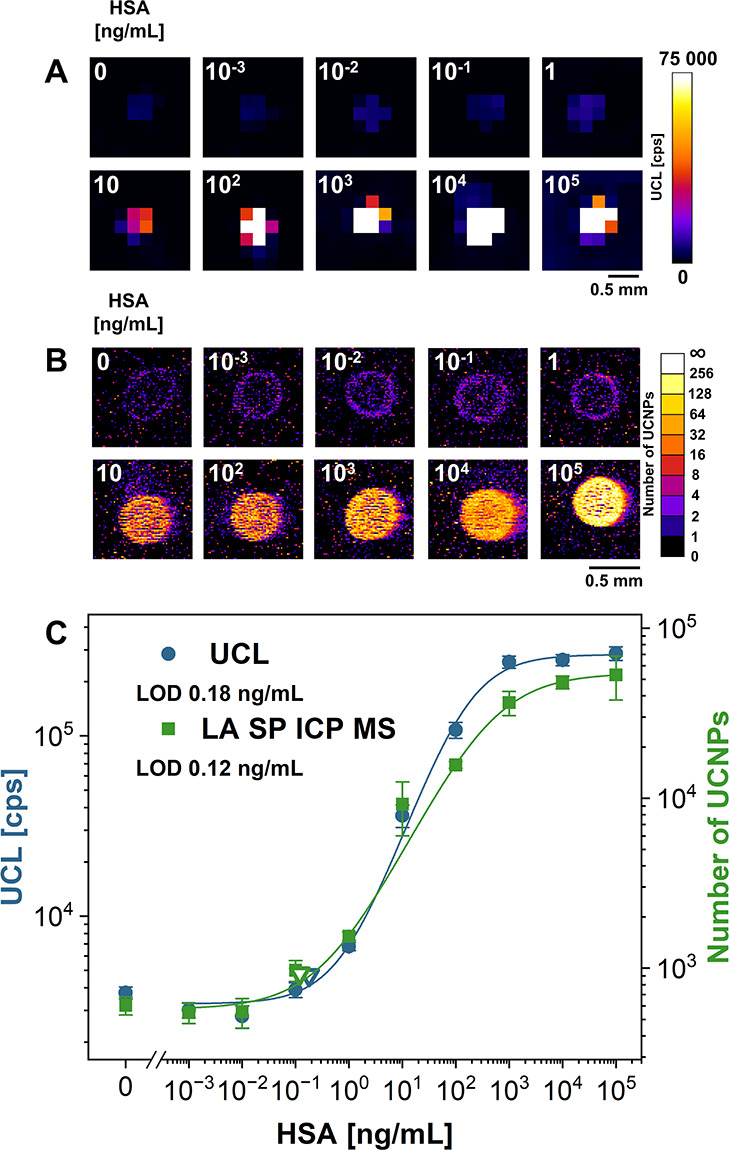
Calibration curves for the determination of HSA utilizing PLISA based on piezo-driven spotting of the capture antibody. Pad images in pseudocolor scale obtained for (A) UCL intensity readout and (B) UCNP counting by LA SP ICP MS. (C) Corresponding UCL and LA SP ICP MS calibration curves. Empty triangles represent LODs; error bars represent standard deviations.

### Optimization of PLISA for PSA Determination

Another clinically relevant protein biomarker, PSA, was detected to verify the versatility of the PLISA. The analysis was done in 50% bovine serum to test the performance of the assay in conditions relevant to the clinical sample analysis (Figure 3A,B). A calibration curve for PSA detection was prepared, reaching an LOD of 2.4 pg/mL for UCL readout (Figure 3C). Although the PSA analysis was carried out in serum, the achieved LOD was significantly lower than in the case of HSA detection in the buffer. This is caused by using different antibodies, as immunoassay performance strongly relies on the affinity between the antibody–antigen pair.^32^ These findings agree with our previous work, where a similar trend was observed for the HSA and PSA detection with the same immunoreagents.^4^

**Figure 3:**
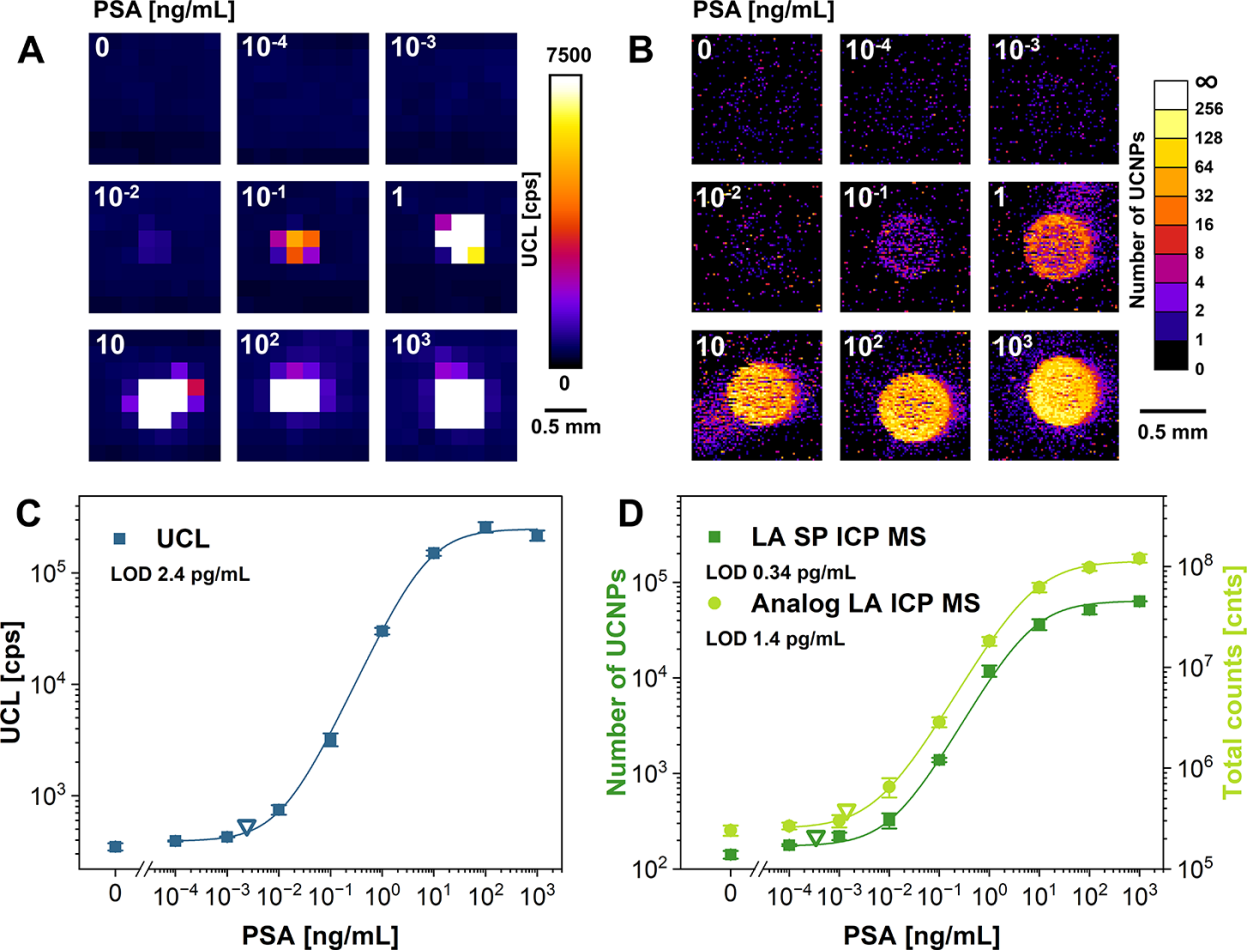
Determination of PSA in serum. Pad images in pseudocolor scale obtained for (A) UCL intensity readout and (B) UCNP counting by LA SP ICP MS. Corresponding calibration curves for (C) UCL and (D) MS detection in analog and digital modes. Empty triangles represent LODs; error bars represent standard deviations.

The LA SP ICP MS readout of the PSA assay in analog and digital mode reached the LODs of 1.4 pg/mL and 0.3 pg/mL, respectively (Figure 3D). Thanks to digital processing, sensitivity improved 5-fold and 8-fold compared to the analog ICP MS and UCL detection, respectively. The key advantage of the digital readout is the ability to filter out analog noise and signals from aggregates (Figure S2B), both contributing to noise that typically deteriorates the assay performance. LA SP ICP MS can estimate the number of UCNPs in aggregates, allowing for their exclusion during the data analysis. In the blank sample detection area of ∼ 0.3 mm^2^, 141 ± 13 UCNPs were detected alongside 4 ± 1 aggregates, ranging from 8 to 17 UCNPs per aggregate, which accounts for ∼ 30% of the signal. In both HSA and PSA analysis, the blank signal is not fully zero due to nonspecific adsorption of assay reagents onto the nitrocellulose surface. The effect was weaker in the PSA analysis, probably due to the higher specificity of the antibodies. At lower analyte concentrations, aggregates contribute proportionally to the increased noise level in analog readouts, and their exclusion in the digital mode ensures more accurate results and lower LOD. Coincidence of two or more UCNPs that becomes more probable at elevated analyte concentrations can be identified by software due to the knowledge of the single UCNP signal from the UCNP peak area histogram mode. Moreover, the upper analyte concentration limit is inherently constrained by the immunoassay itself – at high analyte concentrations, the number of available binding sites becomes limiting, effectively capping the number of detectable UCNPs. Overall, the digital readout based on UCNP counting provides superior analyte quantitation and minimizes instrumental noise.

### Determination of PSA in Clinical Samples

The concentration of PSA in the serum of healthy men typically remains below 4 ng/mL, with PSA levels exceeding this threshold, indicating an elevated risk of prostate cancer.^33^ In addition, when prostate cancer treatment involves radical prostatectomy, the surgical removal of the prostate, PSA levels typically drop to units of fg/mL.^34^ Persistent PSA levels rising above 0.1 ng/mL then indicate significant concerns, such as the presence of residual tumor tissue or cancer recurrence.^35^ Both the UCL and the LA SP ICP MS methods are highly relevant for clinical PSA analysis, as their LODs are in the pg/mL range. Furthermore, these methods enable the precise monitoring of subtle changes in PSA concentrations, which is essential for the early detection of postsurgical complications.

Human serum samples obtained from 15 patients tested for prostate cancer were analyzed to assess the clinical performance of PLISA with UCL and LA SP ICP MS readouts. Serum contains many components that can affect immunoassay performance, such as other proteins, various small organic molecules, and possibly even trace amounts of medications.^36^ All of them can negatively influence assay sensitivity by nonspecific interactions with the nitrocellulose surface and immunoassay reagents.^37^ The achieved sensitivity enabled a reduction of matrix effects through simple dilution; serum samples were diluted 100-fold prior to analysis, which also ensured alignment with the assay working range. The PSA levels obtained by immunoassay with UCL and LA SP ICP MS readout were compared to the reference concentrations measured by the standard electrochemiluminescence immunoassay method. The results of PLISA aligned well with the reference method (Figure 4, Table S1); the slopes of 0.974 and 1.018 and coefficients of determination of 0.75 and 0.69 for UCL and LA SP ICP MS, respectively, demonstrated a strong correlation between the methods. Recovery rates ranged from 78 to 127% for UCL and 76 to 137% for LA SP ICP MS detection. These variations in recovery highlight some limitations, such as the anonymization of clinical samples, which prevented access to patient information, including medication for secondary diseases and other factors that could influence the immunoassay performance. Nevertheless, the results highlight LA SP ICP MS as a highly sensitive readout method, particularly suited for immunoassays for the analysis of clinical samples with low sample volumes and concentrations. Moreover, the strong correlation with the reference method confirmed the applicability of the developed immunoassay for the analysis of complex samples, as other proteins contained in the human serum did not cause significant interference with PSA detection.

**Figure 4:**
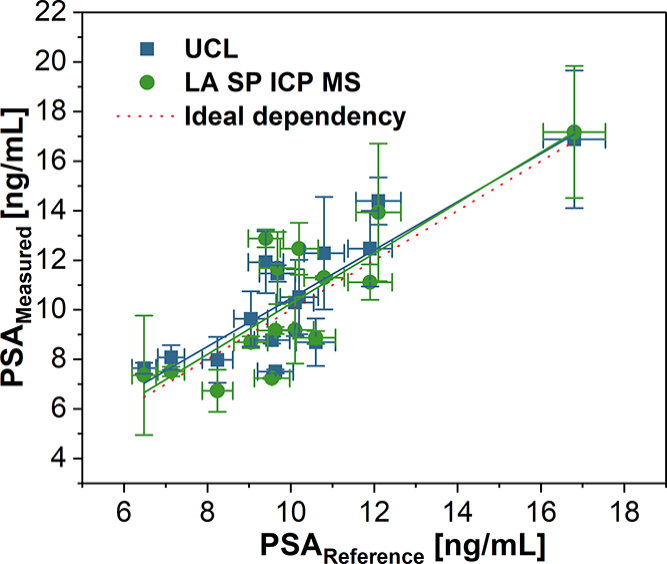
Correlation of PSA concentrations in the clinical samples found by UCL and LA SP ICP MS with the reference electrochemiluminescence immunoassay. Error bars represent standard deviations.

### Comparison with Other Particle-Linked Assays

A range of single-molecule approaches can be utilized to detect biomolecules, as summarized in several reviews.^38–41^ Therefore, for better clarity, we discuss only a comparison with relevant MS-based approaches. A table comparing nanoparticle-based immunoassays with readout based on SP ICP MS and analog LA ICP MS is available in the SI (Table S2). Only a few reports on immunoassays utilize the SP ICP MS readout. Homogeneous SP ICP MS immunoassays rely on changes in NP size and/or concentration in response to analyte concentration. For instance, quantitation of carcinoembryonic antigen (CEA) with an LOD of 0.21 ng/mL was achieved using AuNPs, allowing the method to determine both the count of AuNPs and the size of the formed aggregates.^42^ A similar approach was used for multiplex analysis of CEA (LOD of 0.23 ng/mL, label based on AgNPs), carbohydrate antigen 125 (CA125; LOD of 0.43 U/mL, AuNPs), and Lewis carbohydrate antigen 19–9 (CA19–9; LOD of 0.24 U/mL, PtNPs), where the multielement NP labels were detected.^43^ The advantage of homogeneous immunoassays lies in their rapid, user-friendly “one-pot” reactions, followed by nebulizer ICP MS.

In contrast, heterogeneous immunoassays require the immobilization of antigens on a solid surface. Studies utilizing AuNPs as labels in assays with MTP-based solid phase have been reported. For example, Hu et al.^44^ detected α-fetoprotein in a competitive assay with an LOD of 160 ng/mL, and Liu et al.^45^ developed a sandwich assay for human IgG with an LOD of 0.1 ng/mL. However, before nebulization into the ICP MS, the immunocomplexes had to be released from the solid phase through acid addition or sonication. As an alternative, magnetic particles have been explored as a solid phase for heterogeneous immunoassays and have been employed in wash-free multiplex assays for CEA (LOD of 0.1 ng/mL, AgNP), carbohydrate antigen 72–4 (LOD of 0.20 U/mL, PtNP), and CA19–9 (LOD of 0.21 U/mL, AuNP) in either sandwich or competitive formats, utilizing unbound NPs for the analysis.^46^ Similarly, Cao et al.^47^ used quantum dots and metal NPs for quantitation of cytokeratin fragment antigen 21–1 (CYFRA21–1; LOD of 0.02 ng/mL, AuNPs), CEA (LOD of 6 pg/mL, ZnSe QDs), and carbohydrate antigen CA15–3 (LOD of 0.25 mU/mL, AgNPs). In another study, ZnSe QDs were utilized in a heterogeneous magnetic particle immunoassay for CEA with an LOD of 6 pg/mL.^48^ A study utilizing MoS_2_ and ZnS quantum dots employed a sandwich immunoassay on magnetic particles for the multiplex detection of myeloperoxidase and osteopontin. This method achieved LODs of 4 pg/mL for myeloperoxidase and 5 pg/mL for osteopontin, making it the most sensitive SP ICP MS immunoassay reported to date.^49^

From the perspective of LA ICP MS, research on its use for dot-blot particle-linked immunoassays is scarce. In the work of Tvrdoňová et al.,^50^ direct dot-blot immunoassay was performed on polyvinylidene fluoride membrane with subsequent analog LA ICP MS readout. The authors achieved an LOD of 22 ng/mL of mouse IgG as a model analyte. A similar dot-blot assay detected p53 protein with an LOD of 2.6 ng/mL in the presence of 12 highly purified proteins.^51^ While direct detection offers simplicity, it often suffers from reduced specificity compared to a sandwich configuration.^52^ Moreover, the analysis in none of these cases was carried out in a complex sample matrix.

In contrast to the nebulizer SP ICP MS assays mentioned above, the sample is directly introduced from the solid phase in PLISA. This ensures significantly higher transport efficiency compared to the analysis of a dispersion.^53^ Importantly, our method does not require releasing immunocomplexes from the solid phase into liquid, further streamlining the process. Both these improvements contribute to lower LODs. Our approach uniquely enables the desorption of intact individual UCNPs and their counting. This capability, combined with the sandwich immunoassay format, allows for achieving considerably lower LODs. The sensitivity depends not only on the readout performance but also on the quality of the employed immunoreagents. Nevertheless, our method significantly improves LOD, exceeding the reported assays by at least 1 order of magnitude, thus demonstrating a substantial enhancement in sensitivity.

## Conclusions

This work introduces a novel type of immunoassay, PLISA, utilizing the LA SP ICP MS readout method. It allows counting individual NP-labeled immunocomplexes from nitrocellulose surface by IR LA of intact UCNPs with SP ICP MS detection. The immunoassay procedure and parameters of LA SP ICP MS readout were first optimized using HSA as a model analyte, reaching LODs of 0.18 ng/mL and 0.12 ng/mL for UCL and LA SP ICP MS readouts, respectively. Under optimal conditions, the detection of PSA in serum was carried out, achieving LODs of 2.4 pg/mL, 1.4 pg/mL, and 0.3 pg/mL for the UCL, analog LA ICP MS, and digital LA SP ICP MS readouts, respectively. The 8-fold and 5-fold improvement of digital readout compared to UCL and LA ICP MS, respectively, clearly demonstrates the superior sensitivity of the approach based on counting individual NP-labeled immunocomplexes. The total analysis time per sample was approximately 3 min for UCL and 15 min for LA SP ICP MS. Digital signal processing allows precise filtering of aggregates of NP labels that could lead to a positive readout error while also reducing noise. Compared to similar methods based on single NP detection or LA ICP MS, the LOD of PLISA is approximately 1 to 6 orders of magnitude lower. Finally, to demonstrate the robustness of our approach, clinical samples from patients screened for prostate cancer were analyzed, showing a strong correlation with the reference electrochemiluminescence immunoassay. This work represents a promising option for ultrasensitive digital readout of immunoassays or microarrays. Moreover, due to the inherent specificity of MS, possible simultaneous detection of labels containing different elements opens doors to extensive multiplex analysis, which will further contribute to the development of more effective biomarker analysis and diagnostic technologies.

## Supplementary Material



## Notes

List of chemicals, information about clinical serum samples, protocols for the synthesis and characterization of UCNP-SA labels, biotinylation of anti-HSA polyclonal antibody, details on the UCL readout, along with LA ICP MS instrumentation details, data evaluation, and statistical analysis of UCL and LA SP ICP MS data. Determination of NP transport efficiency, characterization of UCNPs by LA SP ICP MS, and optimizations of immunoassay for the detection of HSA. Table of PSA concentrations determined in clinical samples and performance comparison with other particle-linked assays with ICP MS readout (PDF)
