# Supplementary material for: Digital Immunoassay for Biomarker Detection Based on Single-Particle Laser Ablation ICP MS
Source: Anal Chem. 2025 Jun 25;97(26):13832–9. doi: 10.1021/acs.analchem.5c00641 (PMC12242906; doi:10.1021/acs.analchem.5c00641)
Supplement: Supplementary file 1 [file ac5c00641_si_001.pdf]

Supporting Information

Digital Immunoassay for Biomarker Detection Based on Single-Particle Laser Ablation ICP MS

Vilém Svojanovský,[1,#] Jakub Máčala,[2,#] Antonín Hlaváček,[3] Aleš Čermák,[4] Jaromír Stráník,[1] Pavel Bouchal,[2] Ivana Mašlaňová,[5] Petr Skládal,[2] Zdeněk Farka,[2,*] Jan Preisler[1,*]

[1] Department of Chemistry, Faculty of Science, Masaryk University, Kamenice 5, 625 00 Brno, Czech Republic

[2] Department of Biochemistry, Faculty of Science, Masaryk University, Kamenice 5, 625 00 Brno, Czech Republic

[3] Institute of Analytical Chemistry of the Czech Academy of Sciences, Veveří 97, 602 00 Brno, Czech Republic

[4] Urology Clinic, University Hospital Brno, Jihlavská 20, 625 00 Brno, Czech Republic

[5] Department of Experimental Biology, Faculty of Science, Masaryk University, Kamenice 5, 625 00 Brno, Czech Republic

# The authors contributed equally.

* Corresponding author. E-mail: farka@mail.muni.cz (Z.F.), preisler@chemi.muni.cz (J.P.)

Table of Contents

1 Materials and Methods S-3

1.1 Chemicals and materials S-3

1.2 UCNP Synthesis S-3

1.3 UCNP Surface Modification S-5

1.4 UCNP Characterization S-6

1.5 Biotinylation of Polyclonal anti-HSA Antibody S-6

1.6 LA SP ICP MS instrumentation S-6

1.7 Luminescence Readout S-7

1.8 Statistical analysis of UCL and LA SP ICP MS data S-8

2 Results and Discussion S-9

2.1 Characterization of UCNP labels S-9

Figure S1: Characterization of UCNP labels S-9

2.2 Determination of NP Transport Efficiency of the LA SP ICP MS S-9

2.3 SP ICP MS Characterization of UCNPs and AuNPs S-11

Figure S2: SP ICP MS characterization of UCNPs S-11

2.4 Optimization of Immunoassay for the Detection of HSA S-12

Figure S3: UCL images of whole pads coated with the antibody for HSA detection S-12

Figure S4: Optimization of antibody coating and LA SP ICP MS readout S-13

Figure S5: LA SP ICP MS readout of HSA detection utilizing four single-line scans S-14

Figure S6: Optimization of different whole-pad coating approaches S-15

Figure S7: HSA detection utilizing coating of a smaller area of the pads S-16

Figure S8: Optimization of piezo-driven dispenser antibody deposition S-17

Table S1: PSA determination in clinical samples by UCL and LA SP ICP MS S-18

Table S2: Comparison table of related assays S-19

3 References S-20

1 Materials and Methods

1.1 Chemicals and Materials

Chemicals and Materials. Tween 20, HSA, anti-HSA polyclonal antibody (A0433), and bovine serum were purchased from Merck (Germany). Recombinant PSA (ab283430) and monoclonal anti-PSA antibody (ab403) were obtained from Abcam (UK), biotinylated anti-PSA polyclonal antibody (BAF1344) from Bio-Techne (USA), and anti-HSA monoclonal antibody (AL-01) from Exbio (Czech Republic). SuperBlock TBS (SB) and Protein-Free blocking buffer PBS (PFB) were obtained from Thermo Fisher Scientific (USA). Oncyte Nova nitrocellulose film slides (64 nitrocellulose pads) and ProPlate 64-well slide gaskets were purchased from Grace Bio-labs (USA). Other chemicals were purchased from Penta (Czech Republic), P-LAB (Czech Republic), Carl Roth (Germany), or Merck (Germany).

Buffers included phosphate-buffered saline (PBS; 10 mM NaH_2PO_4/Na_2HPO_4; 2.7 mM KCl; 137 mM NaCl; pH 7.4), coating buffer (50 mM $NaHCO_3/Na_2CO_3$, 0.05% NaN_3; pH 9.6), washing buffer (50 mM Tris, 150 mM NaCl, 0.05% NaN_3, 0.05% Tween 20; pH 7.5), blocking buffer (20% SB and 20% PFB in WB), and assay buffer (50 mM NaH_2PO_4/Na_2HPO_4, 150 mM NaCl, 1 mM KF, 0.1% PEG_{6000}, 0.02% Tween 20, 0.05% NaN_3, 10% PFB; pH 7.5).

Clinical samples of human serum for PSA analysis were obtained from 15 patients tested for prostate cancer with written consent from all participants; the study was approved by the Ethics Committee of the University Hospital Brno (project number 24/22). The reference PSA concentrations were measured using the Elecsys electrochemiluminescence immunoassay analyzer (Roche, Germany).

1.2 UCNP Synthesis

Photon-upconversion nanoparticles (UCNPs) with a composition of $NaYF_4:Yb^{3+},Tm^{3+}$ (Y:Yb:Tm ratio of 80%:18%:2%) were prepared utilizing the high-temperature co-precipitation method previously described in our published protocols.[1,2]

Synthesis of trifluoroacetate precursor

Y_2O_3 (1355 mg, 6 mmol), Yb_2O_3 (532 mg, 1.35 mmol), and Tm_2O_3 (57.9 mg, 0.15 mmol) were put into 250 mL round-bottom flask together with 12 mL of trifluoroacetic acid (TFA) and 12 mL of water. The mixture was heated under the reflux until all the oxides were dissolved completely. Afterward, the mixture was left to cool to room temperature (RT). Then, $NaHCO_3$ (1.26 g, 15 mmol) was slowly added, followed by heating to 110 °C overnight to let all the TFA and water evaporate. The obtained white powder of trifluoroacetate precursors was mixed with oleic acid (45 mL, 40.3 g), octadec-1-ene (45 mL, 35.5 g), sodium oleate (822 mg, 2.7 mmol), and methanol (30 mL). The reaction mixture was boiled at 110 °C under a nitrogen

atmosphere for 20 min. Subsequently, the flask was cooled to RT, and nitrogen flow was used to remove all the air from the inside of the flask.

Synthesis of seed nanoparticles

$YCl_3 \times 6\ H_2O$ (874 mg, 2.88 mmol), $YbCl_3 \times 6\ H_2O$ (251 mg, 0.648 mmol), and $TmCl_3 \times 6\ H_2O$ (27.6 mg, 0.072 mmol) were dissolved in 30 mL of methanol in a 250 mL three-neck round-bottom flask. Then, 24.2 g (27 mL) of oleic acid and 49.7 g (63 mL) of octadec-1-ene were added, and the mixture was heated to 170 °C under a nitrogen atmosphere until the evaporation of all the volatile liquids was completed. The temperature was let to decrease to 50 °C, and the nitrogen atmosphere was disconnected. Then, the solution of NH_4F (533 mg, 14.4 mmol) and NaOH (360 mg, 9 mmol) in 30 mL of methanol was added to the three-necked flask, followed by connecting the protective nitrogen atmosphere and stirring for 30 min. The temperature was carefully increased to 150 °C, allowing all the methanol to evaporate. After 10 min, the temperature was rapidly increased at a rate of ~ 10 °C per minute until reaching the final temperature of 300 °C. The reaction mixture was kept at 300 °C for 90 min under the nitrogen flow, followed by stopping the heating and spontaneous cooling of the reaction mixture down to RT. The prepared UCNPs were precipitated using 180 mL of propan-2-ol and centrifuged for 10 min at 1000 *g* to collect the pellet of UCNPs. The supernatant was discarded, and the pellet was washed with 90 mL of methanol, followed by dispersing the pellet utilizing ultrasonication. The dispersion was then centrifuged for 10 min at 1000 *g*, followed by dispersing the obtained pellet in 20 mL of cyclohexane. Then, 100 mL of methanol were added to precipitate the UCNPs. The precipitate was redispersed in 30 mL of cyclohexane and centrifuged for 20 min at 50 *g* to remove any solid contaminants from the final seed UCNP dispersion that was subsequently used to grow larger nanoparticles.

Nanoparticle growth

The larger UCNPs were prepared by growing the seed UCNPs by the addition of trifluoroacetate precursors. Dispersion of seed UCNPs in cyclohexane was inserted into the three-necked round-bottom flask, followed by the addition of oleic acid (5.5 mL, 4.9 g), octadec-1-ene (17 mL, 13.4 g), sodium oleate (207 mg, 0.68 mmol), and methanol (20 mL). The mixture was heated to 150 °C under the nitrogen atmosphere, and the temperature was kept for 30 min, followed by a rapid increase to 300 °C at the rate of 10–15 °C/min. The trifluoroacetate precursors were injected with a syringe in 10 min intervals between the additions. The volumes of added precursors were 3.5, 4.0, 4.6, 5.2, 6.0, 6.8, 7.8, 8.9, 10.2, and 11.6 mL. After the last addition, the mixture was boiled at 300 °C for another 10 min and then cooled to RT. The synthesized UCNPs were precipitated by 190 mL of propan-2-ol and collected by centrifugation for 10 min at 1000 *g*. Then, the pellet was washed with methanol, centrifuged as in the previous step, and dispersed in 60 mL of cyclohexane. The dispersion was centrifuged for 20 min at 50 *g* to remove potential large contaminants from the final nanoparticle dispersion.

1.3 UCNP Surface Modification

Surface modification of UCNPs was carried out according to our previously published protocols.[3–5]

Alkyne-PEG-neridronate linker was prepared as previously described.[6] First, 30 mg of neridronate (Merck, Germany) were dissolved in 128 µL of 1 M aqueous solution of NaOH, followed by the addition of 398 µL of phosphate buffer (PB; 50 mM NaH_2PO_4/Na_2HPO_4; pH 7.6) and sonication of the mixture for 5 min. Then, 500 µL of 15 mM α-*N*-hydroxysuccinimide-ω-alkyne polyethylene glycol (M_W 3000 Da; Rapp Polymere, Germany) in PB was added. The mixture was incubated with shaking for 2 h at RT and then overnight at 4 °C. Afterward, the mixture was purified by dialysis against distilled water for 5 days (exchange of water 3 times a day), utilizing the Spectra/Por Float-A-Lyzer G2 dialysis tube (500–1000 Da MWCO; Carl Roth, Germany). The purified solution was pipetted into a glass vial, lyophilized (Alpha 1-2, Christ, Germany) for 24 h, and stored at 4 °C.

To bind the alkyne-PEG-neridronate linker to the UCNP surface, 10 mg of UCNPs dispersed in the 500 µL of cyclohexane were mixed with 500 µL of 200 mM HCl. The mixture was then incubated with shaking at 38 °C for 40 min and sonicated for 30 min, resulting in the exchange of the surface oleic acid ligands for Cl^- ions, allowing the phase transfer of UCNPs from organic solvent to water. The water phase was transferred to another tube, excess acetone was added, and the mixture was centrifuged for 1 h at 1000 *g*. Then, the supernatant was discarded, and the UCNP pellet was dispersed in 500 µL of distilled water and sonicated for 5 min. Alkyne-PEG-neridronate linker (3 mg) was dissolved in 500 µL of distilled water, added to the UCNPs, and the mixture was incubated overnight at 38 °C with shaking. The conjugate of UCNPs with the linker was purified by dialysis against distilled water containing 1 mM KF for 3 days (exchange of solution 3 times a day), utilizing the Float-A-Lyzer G2 dialysis tube (50 kDa MWCO).

Prior to conjugation with UCNPs, streptavidin (Thermo Fisher Scientific, USA) was modified with an NHS-dPEG$_8$-azide linker (Merck, Germany). First, 31.25 µL of 200 mM NHS-dPEG$_8$-azide in DMF were added to 150 µL of 4 mg/mL streptavidin in PB. Then, 187.5 µL of PB were added, and the reaction mixture was incubated for 2 h at RT with shaking. The reaction was terminated by the addition of 50 µL of washing buffer (50 mM Tris, 150 mM NaCl, 0.05% NaN_3, 0.05% Tween 20; pH 7.5). Afterward, streptavidin-azide conjugates were purified using the centrifugation filtration (Amicon Ultra, 10 kDa MWCO; Merck, Germany) and stored at 4 °C in PB in a final concentration of 1 mg/mL.

Finally, for the conjugation of UCNPs with streptavidin-azide, 100 µL of Tris buffer (375 mM Tris; pH 7.5), 10 µL of 25 mM $CuSO_4$, and 10 µL of Tris(3-hydroxypropyltriazolylmethyl)amine (Merck, Germany) were mixed and added to 10 mg of alkyne-PEG-neridronate-UCNPs dispersed in 1.4 mL of distilled water with 1 mM KF, and the mixture was purged with argon for 40 min to remove the dissolved oxygen. Then, 100 µL of streptavidin-azide (1 mg/mL in PB) were added, and the mixture was purged for another 10 min. The click reaction was started by the addition of 10 µL of 20 mg/mL sodium ascorbate

(Merck, Germany) in distilled water. The mixture was again purged with argon for 2 h. The UCNP-SA conjugates were purified by dialysis in the Float-A-Lyzer G2 dialysis tubes (100 kDa MWCO) against the dialysis buffer (50 mM Tris, 0.05% NaN_3, 1 mM KF; pH 7.5) at 4 °C for 3 days (exchange of fresh buffer 3 times a day). The purified UCNP-SA conjugates were stored at 4 °C.

1.4 UCNP Characterization

UCNPs were characterized using transmission electron microscopy (TEM) and dynamic light scattering (DLS). For the TEM analysis, 5 µL of UCNP dispersion in cyclohexane were dispensed on a carbon-coated (12-nm continual foil) copper grid and left to dry at RT. TEM images were acquired by Talos F200C (Thermo Fisher Scientific, Czech Republic). The size of individual particles was analyzed in ImageJ software (National Institutes of Health, USA).[7]

Hydrodynamic diameters of UCNPs and their conjugates were analyzed by DLS, using the Zetasizer Nano ZS (Malvern, UK). The particles were diluted to a concentration of 20 µg/mL in cyclohexane in the case of oleic acid-capped UCNPs or dialysis buffer in the case of alkyne-PEG-neridronate-UCNPs and UCNP-SA conjugates. Measurements were carried out at 25 °C with the scattering angle of 173°. Zeta potential of UCNP-SA conjugate was measured using Zetasizer Nano ZS. The particles were diluted to a concentration of 0.5 mg/mL in a 0.1× PBS buffer (5 mM NaH_2PO_4/Na_2HPO_4, 15 mM NaCl, pH 7.4); the measurement was conducted at 25 °C.

1.5 Biotinylation of Polyclonal anti-HSA Antibody

NHS-LC-biotin (Merck, Germany) was dissolved in dry DMF to a concentration of 20 mg/mL, and 1.67 µL of the solution was added to 100 µL of the polyclonal anti-HSA antibody (10.9 mg/mL in PB). The mixture was shaken for 10 min at RT, followed by the addition of another 1.67 µL of NHS-LC-biotin. The incubation was carried out for 2 h at RT, followed by overnight at 4 °C, both with shaking. The biotinylated antibody was purified by centrifuge filtration using the Amicon Ultra centrifugation filters (100 kDa MWCO); the purified antibody was diluted with PB to a final concentration of 2 mg/mL and stored at 4 °C.[2]

1.6 LA SP ICP MS Instrumentation

The 2940 nm laser beam was guided from the source into a 4× Galilean beam expander composed of an anti-reflective (AR)-coated CaF_2 plano-convex lens with a 12.7 mm diameter and −25.0 mm focal length and an AR-coated CaF_2 plano-convex lens with 25.4 mm diameter and 100.0 mm focal length. Afterward, the beam was reflected by 90° using a silver mirror in an optical cube, followed by reduction by an iris to a top-hat near profile (all from Thorlabs, USA). The beam was focused on the sample using an AR-coated aspheric plano-convex lens with 8.00 mm diameter and 5.95 mm focal length (Edmund Optics, USA) to a diameter of 22 µm and a fluence of 11.4 J/cm^2.

The ablation cell was operated in two configurations: standard and fast washout. In the standard configuration, the cell dimensions above the slide were 76.1 mm × 26.1 mm × 3.0 mm. Helium served as the carrier gas at a flow rate of 1.0 L/min, with an additional 1.0 L/min of argon introduced after the cell exit. The fast washout configuration featured a rectangular main channel above the slide, measuring 76.1 mm × 4.7 mm × 3.0 mm. In this setup, helium flowed at 1.6 L/min and 0.4 L/min of argon. For maximum transport efficiency, all laser ablations were performed on the half of the glass slide positioned closer to the outlet of the ablation cell.[8] To ablate the samples from the second half, the slide was rotated 180° to maintain LA conditions as consistent as possible.

The laser spot position was set by the XY motion of the cell with 8MT-175-150 and 8MT-175-100 stages (both from Standa, Lithuania). The stages were controlled using a program developed in LabVIEW (National Instruments, USA). The laboratory-built shutter was employed to block the laser beam during the returning phase of the flyback raster mode. In this mode, the beam scans from right to left and from top to bottom; it returns to the start of a new line without collecting data. A stainless steel capillary tube with a 1.34 mm internal diameter (Swagelok, USA) was used for aerosol transport. Steel capillary was connected to a laser ablation adapter (31-808-4034, Glass Expansion, Australia) and flowed into a plasma torch with a 2.5 mm injector (G3280-80053; AHF, Germany). The sample aerosol was introduced into a quadrupole mass spectrometer (ICP MS 7900, Agilent, USA) through nickel sampler and skimmer cones (G3280-67040, G8400-67200; AHF, Germany). The ICP operated at 1550 W was sustained by 15 L/min of Ar plasma gas and 0.9 L/min of Ar auxiliary gas. The plasma torch position and ion optics voltage settings were optimized using a MicroMist nebulizer equipped with a Scott spray chamber. A 1 ppb yttrium solution in 2% HNO_3 served as the tuning standard (Analytika, Czech Republic).

1.7 Luminescence Readout

The UCL of UCNP labels was measured using an UPCON S-Pro reader (Labrox, Finland). The UCNPs were excited using a 980 nm laser with a 976/30 nm excitation filter, and the emission was collected using a photomultiplier with an 810/40 nm emission filter and a D900 dichroic mirror. The nitrocellulose pads were scanned with a step size of 0.5 mm and an integration time of 500 ms for individual points. The obtained data were imported into ImageJ software, and pad images were reconstructed utilizing the pseudocolor intensity scale. Detection spots were localized in the images, and their average intensities were calculated.

1.8 Statistical Analysis of UCL and LA SP ICP MS data

For both the UCL and ICP MS, the data were statistically evaluated using the OriginPro 2023 software (OriginLab, USA). For each analyte concentration, the mean and standard deviation were calculated from the intensity values or number of UCNPs in four replicate pads for UCL and LA SP ICP MS, respectively. Subsequently, the data were fitted with a four-parameter logistic function:

$$Y = \frac{A_1 - A_2}{1 + \left(\frac{c}{EC_{50}}\right)^s} + A_2 \quad \text{(S1)}$$

where Y represents the signal intensity of UCL and analog LA ICP MS or the number of UCNPs for the LA SP ICP MS, A_1 represents the sigmoidal curve minimum corresponding to the assay background, A_2 is the sigmoidal curve maximum, c is the analyte concentration, EC_{50} is the half-maximal effective concentration, and s is the slope at the inflection point.

The LODs were calculated from the regression curves as concentrations corresponding to the Y_{LOD} value:

$$Y_{\mathrm{LOD}} = A_1 + 3S_{\mathrm{B}} \quad \text{(S2)}$$

where A_1 is the background value obtained from the logistic fit, and S_{B} represents the standard deviation of the blank. The signal-to-background (S/B) ratios were calculated using the signals obtained for the respective analyte concentration and the corresponding blank. The standard deviations of the S/B values were calculated considering the propagation of uncertainties. Working ranges were estimated as the intervals between the EC_{20} and EC_{80} of the four-parameter logistic curve.

2 Results and Discussion

2.1 Characterization of UCNP Labels

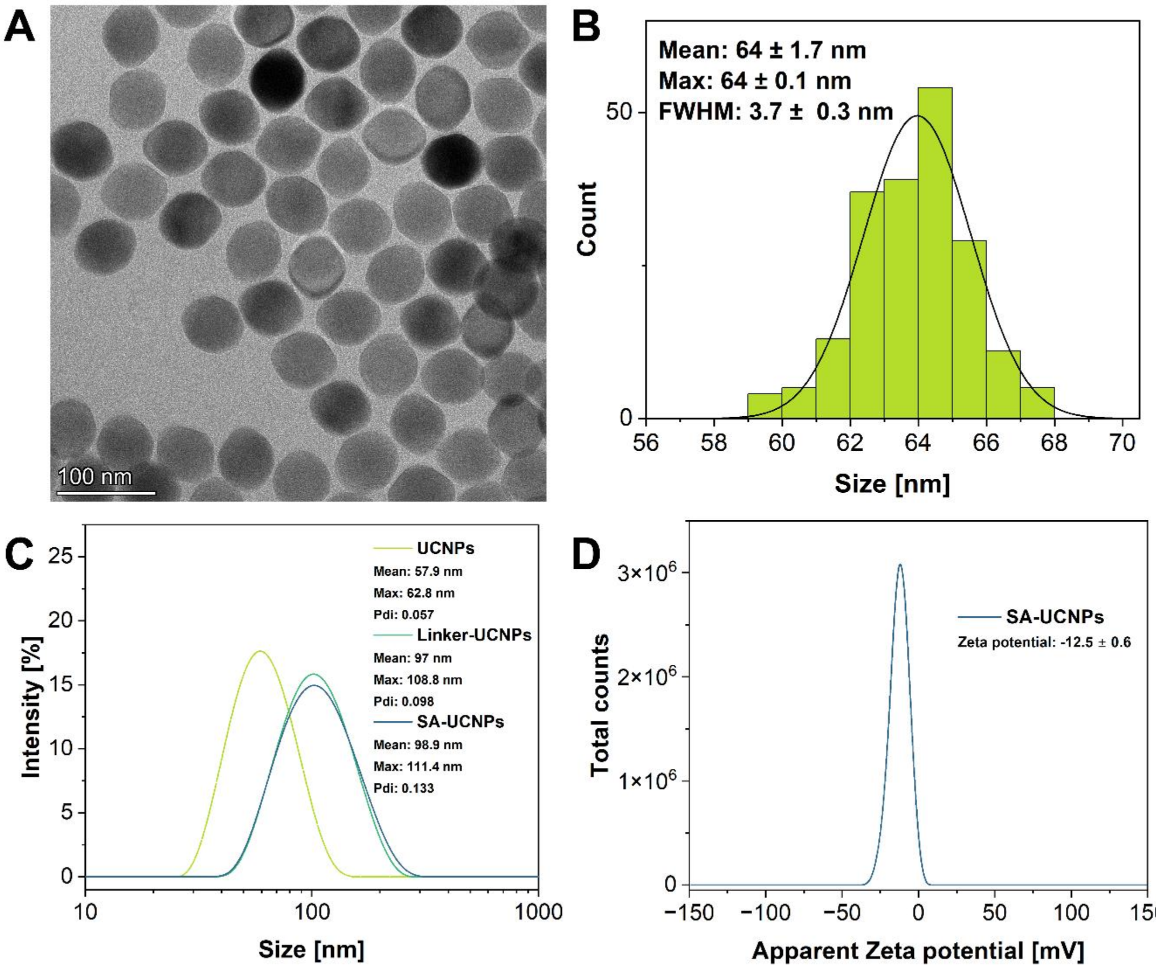

Figure S1. Characterization of the UCNP labels. (A) TEM image of oleic acid-capped UCNPs and (B) histogram of particle sizes with Gaussian fit (n = 200). (C) Intensity-based measurement of DLS particle size distributions of oleic acid-capped UCNPs, alkyne-PEG-neridronate-UCNPs (Linker-UCNPs), and UCNP-SA bioconjugates. (D) Zeta potential measurement of the SA-UCNP bioconjugate. The B-spline function was used to connect the DLS and zeta potential measurement data. Max represents the x-value of the peak maximum, and Mean represents the average of the longest diameter of the UCNPs in the TEM or the average hydrodynamic diameter of the UCNPs in the DLS.

2.2 Determination of NP Transport Efficiency of the LA SP ICP MS

A stock dispersion of ultra-uniform 50 nm AuNPs functionalized with PEG-carboxyl groups (nanoComposix, USA) was diluted 1400-fold to a final concentration of 35.7 µg/L by pipetting 1 µL of the stock dispersion into 1399 µL of 2 mM sodium citrate buffer (pH 7.9). This dilution was calculated to yield an estimated concentration of ~ 5800 AuNPs per droplet. It should be noted that this value is approximate and may vary between experiments due to pipetting errors,

adsorption, dispersion age, etc. To ensure maximal consistency, the dilution was performed in a single step, and all samples were prepared from the same diluted dispersion at the same time.

For sample preparation, 200 nL of the diluted dispersion was deposited contactless onto gelatin sections using a 500 nL precision syringe (Hamilton, USA). The dispersion was manually agitated prior to each deposition to maintain maximal homogeneity.

The AuNP signals were measured at *m*/*z* of 197 (gold) with a detector frequency of 10 kHz. The plasma torch position and the ion optics voltage settings were optimized using a 1 ppb solution of gold dissolved in 5% HCl (Analytika, Czech Republic). LA SP ICP MS was performed in both configurations of the ablation cell. The number of detected AuNPs was 5230 ± 270 (n = 5) in the fast washout configuration and 5230 ± 190 (n = 4) in the case of the standard configuration. A two-sample t-test was conducted using the following formula:

$$t = \frac{|\bar{x}_1 - \bar{x}_2|}{\sqrt{(n_1-1)s_1^2 + (n_2-1)s_2^2}} \cdot \sqrt{\frac{n_1 n_2 (n_1+n_2-2)}{n_1+n_2}} \qquad \text{(S3)}$$

where $\bar{x}$ corresponds to the mean, s to the standard deviation, and n to the sample size.

The difference was insignificant at a significance level of p = 0.05, suggesting that widening the ablation cell channel did not notably affect transport efficiency. This observation supports the assumption that the transport efficiency was comparable to the 83% reported in our previous study.[9]

2.3 SP ICP MS Characterization of UCNPs and AuNPs

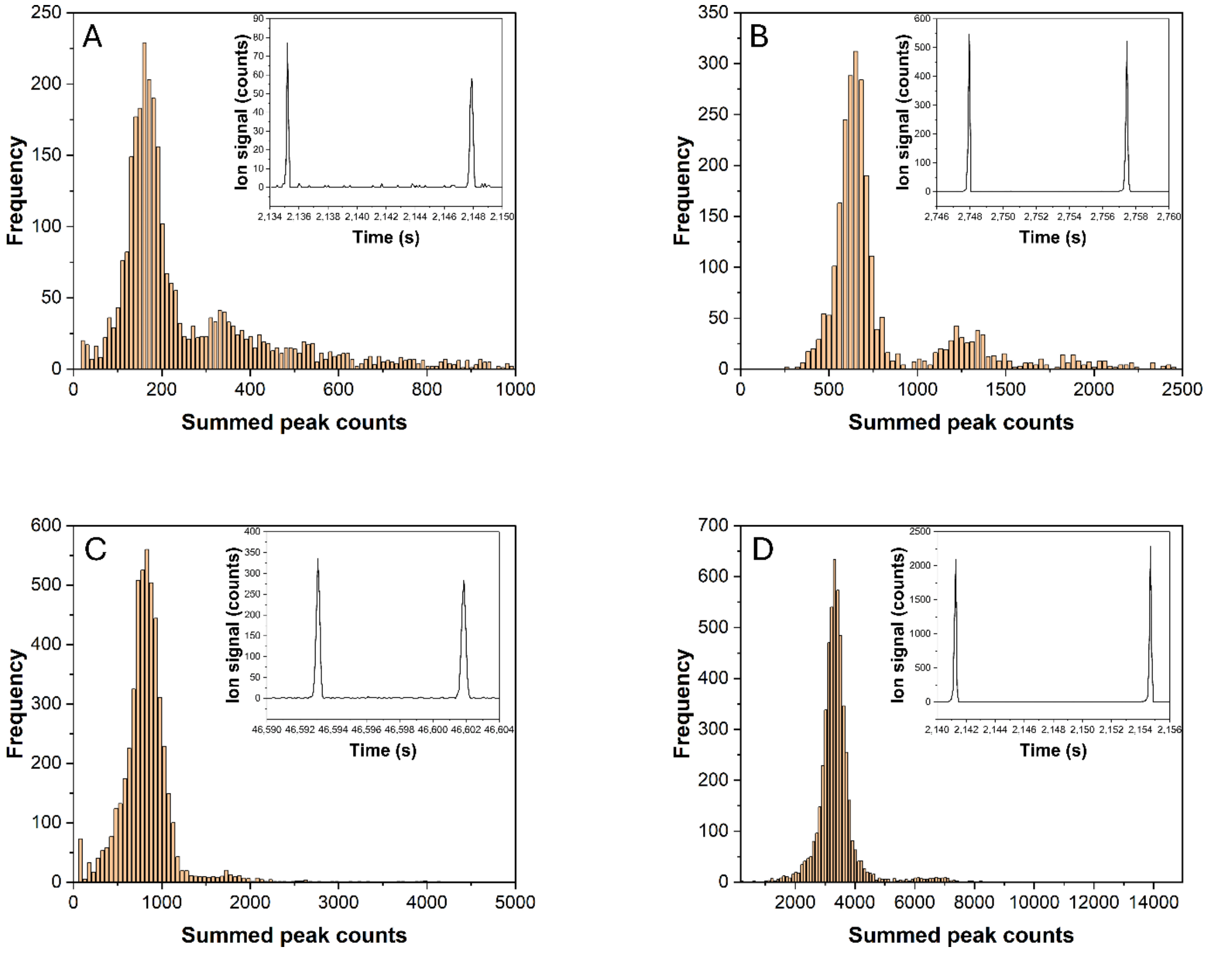

Figure S2. Histograms of yttrium ion signal (*m*/*z* of 89) of (A) nebulizer SP ICP MS of UCNPs diluted to 65 ng/L and (B) LA SP ICP MS of UCNP. Histograms of Au ion signal (*m*/*z* of 197) of (C) nebulizer SP ICP MS of AuNPs diluted to 77.5 ng/L and (D) LA SP ICP MS of AuNP dried droplet.

A notable trend observed in this study is signal intensity shift for NPs introduced via LA compared to nebulizer introduction (**Figure S2**). This phenomenon may be attributed primarily to differences in aerosol composition and behavior within the plasma. During LA, a dry aerosol is introduced, whereas the nebulizer introduces a wet aerosol. In the case of wet aerosol introduction, part of the plasma energy is likely consumed by solvent evaporation, which may reduce the overall plasma temperature and thus decrease the ionization efficiency. Additionally, LA utilizes different gas flow through the injector, which is approximately twice as high as during the nebulization and can significantly alter atomization and ionization equilibria, as well as particle trajectories in the plasma. The gas composition also plays an important role: during LA, a 1:1 Ar/He mixture is introduced into the plasma via the injector, whereas only Ar is used in the nebulization setup. All these factors contribute to the observed differences in atomization and ionization behavior. This effect is also observed for the reference ultra-uniform 50 nm PEG-carboxyl functionalized AuNPs (**Figure S2 C,D**).

2.4 Optimization of Immunoassay for the Detection of HSA

Two 200 µm × 200 µm representative areas, spaced 1 mm apart, were ablated in each nitrocellulose pad (**Figure S3**), and UCNPs were counted. The LODs of 2.1 and 5.8 ng/mL obtained with the UCNP-SA concentrations of 6.5 and 3.25 µg/mL, respectively (**Figure S4A,B**), were worse compared to the luminescence readout (0.2 and 3.4 ng/mL). The higher LODs were attributed primarily to the broad uncertainty in the measurements. Based on the UCL images (**Figure S3**), it is clear that the signal intensity was not homogenous within a single pad. This may be caused by the coffee ring effect or by interactions of coating antibodies with the wall of the gasket used to construct a barrier around the pads for easier incubation and washing. Although scanning the entire reaction site would reduce the influence of signal inhomogeneities across the pad, LA SP ICP MS scans of the whole pads are not practical due to excessive time demands (~ 75 min/pad).

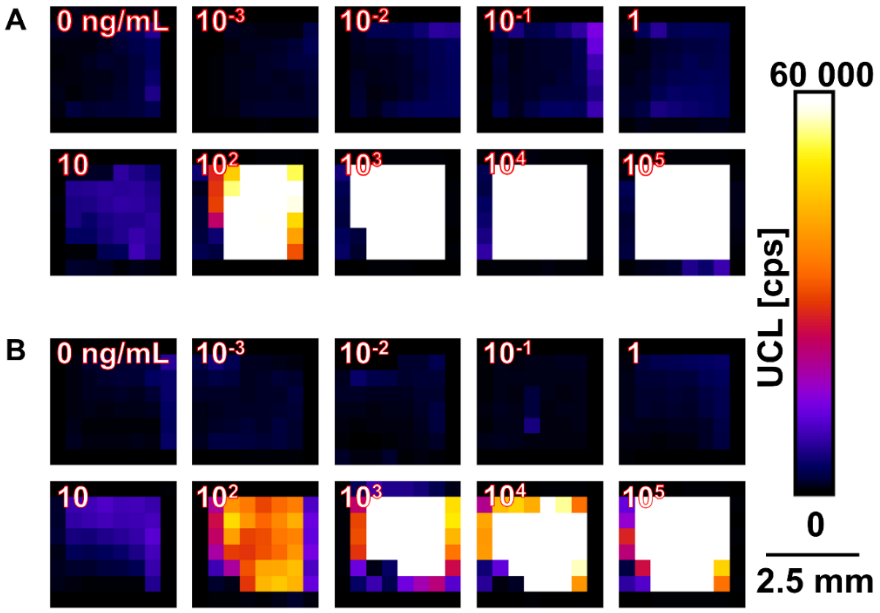

Figure S3. UCL images of whole pads coated with the antibody for HSA detection. The UCNP-SA label concentrations were (A) 6.5 µg/mL and (B) 3.25 µg/mL.

To reduce the impact of coating inhomogeneities, five 200 µm × 200 µm representative areas in a cross configuration, spaced 500 µm apart, were ablated as representative areas of a pad. As this approach covers a larger area, it was hypothesized that it should lead to a lower LOD. The immunoassay was carried out in the same way as in the previous experiment, except for different UCNP-SA conjugate concentrations (6.5 and 13 µg/mL). This adjustment reduced the LODs and confirmed the optimal UCNP-SA concentration of 6.5 µg/mL (**Figure S4 C,D**). However, the obtained LOD of 0.6 ng/mL was still higher compared to the UCL scan (28 pg/mL).

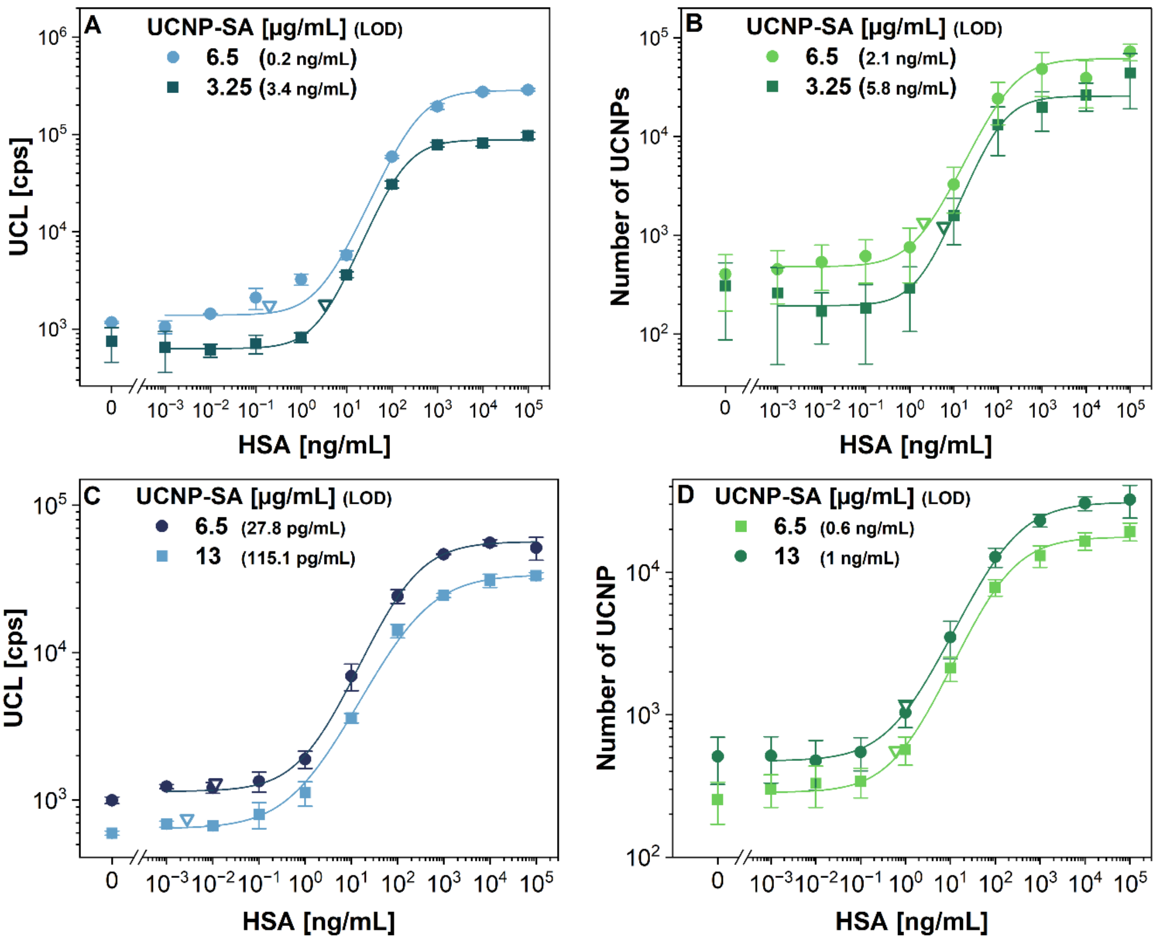

Figure S4. Optimization of antibody coating and LA SP ICP MS readout. Calibration curves for HSA detection obtained with UCNP-SA concentrations of 6.5 and 3.25 µg/mL with corresponding (A) UCL readout and (B) LA SP ICP MS readout based on two 200 µm × 200 µm areas. Calibration curves for HSA detection obtained with UCNP-SA concentrations of 6.5 and 13 µg/mL with corresponding (C) UCL readout and (D) LA SP ICP MS readout based on five 200 µm × 200 µm areas. Empty triangles represent LODs; error bars represent standard deviations.

Thus, a compromise was sought between representative sampling of a pad, accounting for signal gradients, and minimizing the time required for ICP MS analysis. The previous assay was reproduced in the same way, and the approach using four single-line scans was chosen for the ablation (**Figure S5**). To minimize the chance of coincidence in NP detection, the scan speed was reduced to 15 µm/s, as this scanning method avoids overlap of ablation lines. This approach ensures a larger cross-sectional surface area analyzed within each ablation line. Even though this scanning approach offered the potential to cover a large cross-section area within a single pad and was relatively quick (9 min/pad), it did not result in a lower LOD (0.9 ng/mL).

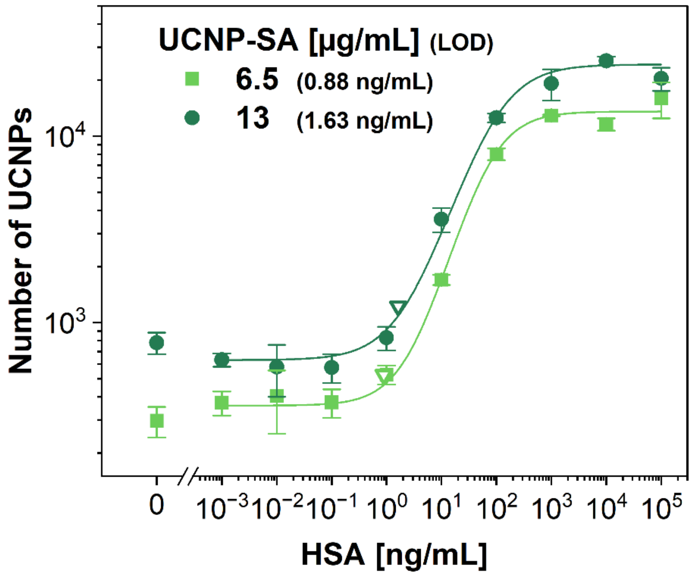

Figure S5. LA SP ICP MS readout of HSA detection utilizing four single-line scans. Empty triangles represent LODs; error bars represent standard deviations.

Although increasing the scanned area improved the sensitivity of the LA SP ICP MS readout, none of the scanning approaches managed to overcome the UCL readout in terms of LOD. This probably stems from signal inhomogeneities across the pads caused by uneven antibody distribution and the partial coffee ring effect formed during the drying of the capture antibody solution in the wells of a gasket, as seen on the UCL scan (**Figure S3**). To further improve the performance of LA SP ICP MS, we hypothesized that reducing the area coated with the capture antibody could allow scanning of the near-entire detection area. Consequently, it could minimize the effect of inhomogeneities in the antibody coating, and improve the performance of LA SP ICP MS detection. Therefore, further optimization of capture antibody deposition was conducted, aiming for the approach yielding the highest *S*/*B* ratio, a prerequisite for high assay sensitivity (**Figure S6**). The results showed that 1 µL of 100 µg/mL AL-01 capture antibody (without surfactants) and conducting the PLISA after only a 30 min incubation of coated capture antibody on nitrocellulose pads yielded the highest *S*/*B* for both the UCL and the LA SP ICP MS, likely because the antibodies had less time to denature. This approach, however, required a minimum volume of 1 µL to maintain pipetting accuracy and did not consistently ensure the homogeneity of the reduced detection area. (~ 2 mm × 2 mm, **Figure S7**). As a result, the performance of the UCL readout improved only slightly, reaching a LOD of 27 pg/mL. However, the performance of LA SP ICP MS improved significantly, reducing the LOD 4 times to 160 pg/mL. The improvement in PLISA performance indicates that reducing the coating inhomogeneities and the possibility of scanning the entire detection area is crucial for lowering the LOD. Therefore, one of the main challenges is ensuring reproducible capture antibody deposition in a small volume over a small area, which can impact the results significantly.

To address this issue, we proposed using an automatic piezo-driven dispenser, which allows depositing the antibody solution on a small area and enables a more reproducible coating, ultimately allowing abandoning the representative area approach that could compromise the precision of ablation measurements. Therefore, various methods of capture antibody deposition were compared. The *S*/*B* ratios were used for comparison; LODs were not determined in this optimization experiment, as only three analyte concentrations and a blank sample were tested. The deposition methods included placing 4×25 droplets in the same spot, 1×25 droplets in the same spot, 4×25 droplets in a square-like pattern in the center of the pad, and 4×25 droplets in the pad corners (**Figure S8**). To compensate for the substantial decrease in the deposited volume compared to the manual deposition, the coating antibody concentration was increased to 500 µg/mL. The number of UCNPs was obtained by counting only those within the defined detection areas. The highest *S*/*B* ratio was achieved for the spotting configuration of 4×25 droplets in the exact location, creating a detection area with a diameter of ~ 600 µm that can be ablated entirely. Therefore, the antibody deposition with a piezo-driven dispenser utilizing repeated spotting to the same location was chosen for further experiments.

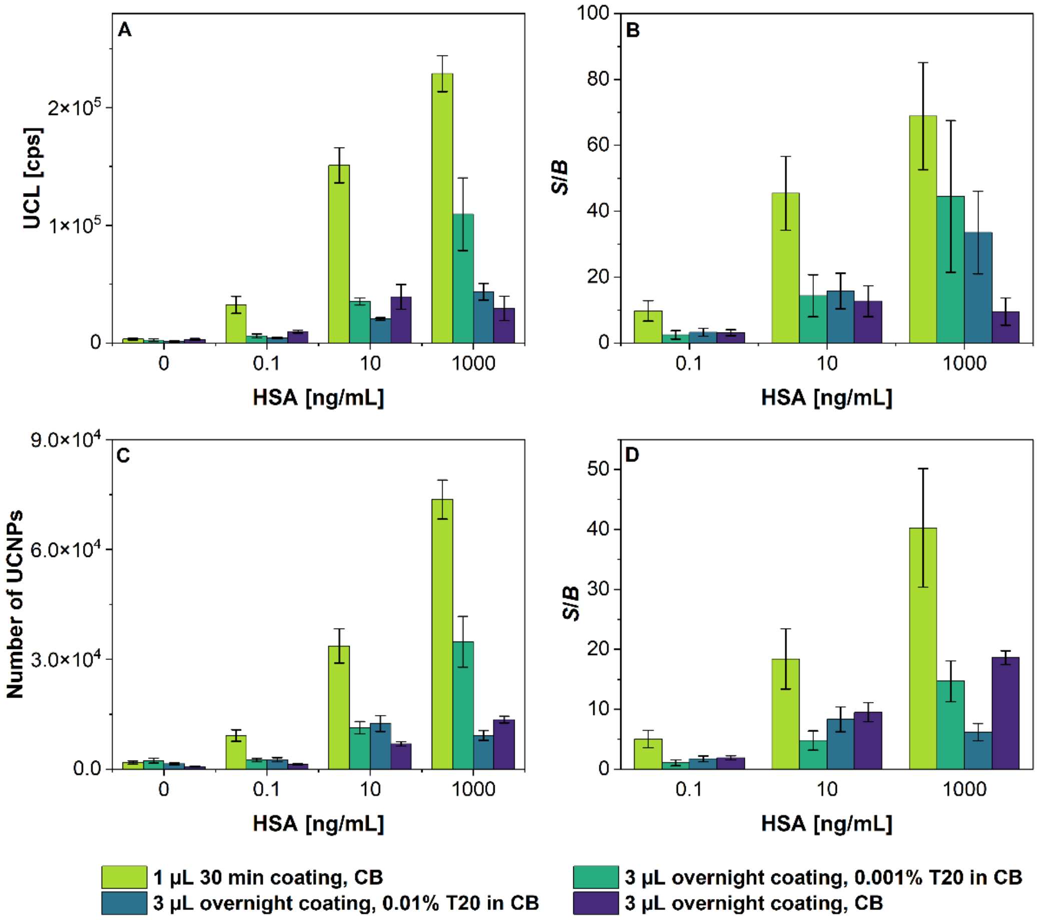

Figure S6. Optimization of whole-pad coating approach. Concentration of AL-01 antibody 100 µg/mL. (A) UCL readout with (B) corresponding *S*/*B* ratios and (C) LA SP ICP MS readout with (D) corresponding *S*/*B* ratios. Error bars represent standard deviations.

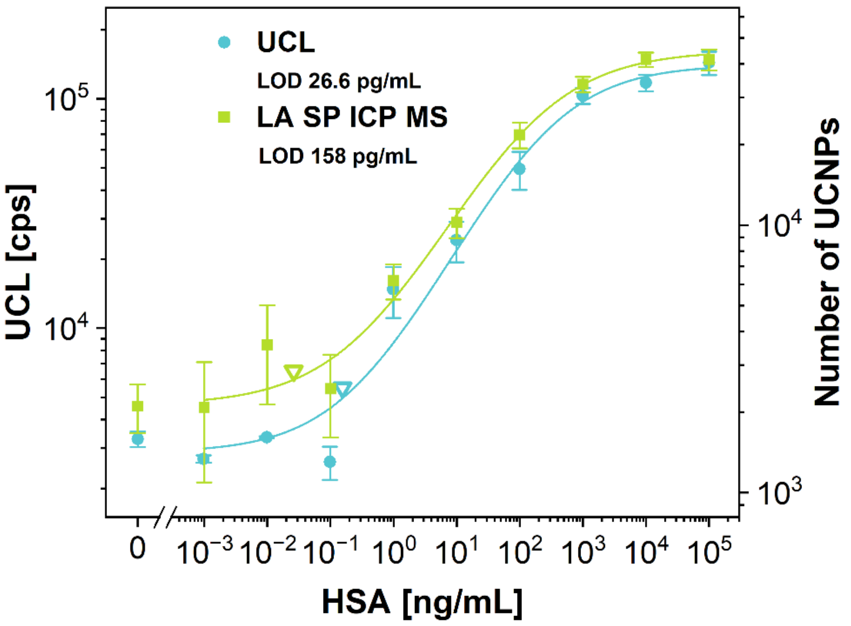

Figure S7. HSA detection in an assay based on coating of a smaller area of the pads. Empty triangles represent LODs; error bars represent standard deviations.

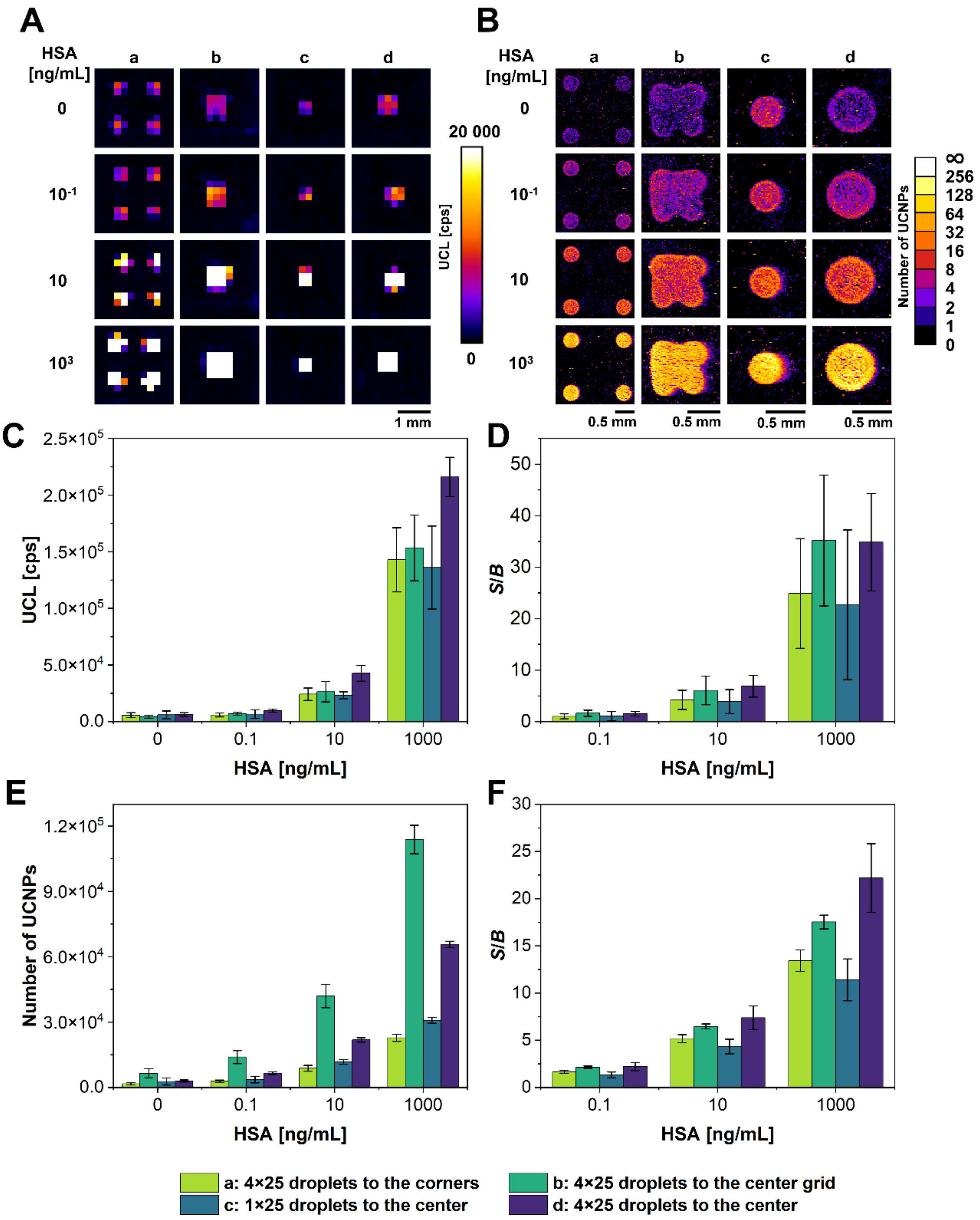

Figure S8. Optimization of piezo-driven dispenser antibody deposition. Concentration of AL-01 antibody 500 µg/mL. Pad images of different coating approaches in pseudocolor scale, and scanned with (A) UCL and (B) LA SP ICP MS. (C) UCL readout with (D) corresponding *S*/*B* ratios and (E) LA SP ICP MS readout with (F) corresponding *S*/*B* ratios. The data shown in panels C to F were calculated from raw signal values. Error bars represent standard deviations. Tested deposition patterns included: (a) 4×25 droplets in the corners of the nitrocellulose pads, (b) 4×25 droplets in the corners of the square grid in the center of the pads, (c) 1×25 droplets in the same spot, and (d) 4×25 droplets in the same spot.

Table S1. PSA determination in clinical samples by UCL and LA SP ICP MS. Electrochemiluminescence immunoassay for total PSA detection (Roche Elecsys) was used as a reference method.

Sample number	Reference [ng/mL]	UCL		LA ICP SP MS	
		Found [ng/mL]	Recovery [%]	Found [ng/mL]	Recovery [%]
1	6.5 ± 0.2	7.6 ± 0.2	118 ± 3	7.4 ± 2.4	114 ± 37
2	8.2 ± 0.3	8.0 ± 0.9	97 ± 11	6.7 ± 0.8	82 ± 10
3	12.1 ± 0.4	14.4 ± 1.0	119 ± 8	13.9 ± 2.8	115 ± 23
4	10.2 ± 0.3	10.5 ± 1.5	103 ± 15	12.5 ± 1.0	122 ± 10
5	9.0 ± 0.3	9.6 ± 1.1	107 ± 12	8.7 ± 0.2	96 ± 3
6	10.1 ± 0.3	10.3 ± 1.3	102 ± 13	9.2 ± 1.3	91 ± 13
7	9.6 ± 0.3	7.5 ± 0.1	78 ± 1	9.2 ± 0.1	95 ± 1
8	9.6 ± 0.3	8.8 ± 0.1	92 ± 2	7.2 ± 0.03	76 ± 1
9	9.4 ± 0.3	11.9 ± 1.2	127 ± 22	12.9 ± 0.4	137 ± 4
10	7.1 ± 0.2	8.1 ± 0.5	113 ± 7	7.5 ± 0.2	105 ± 3
11	10.6 ± 0.3	8.7 ± 1.0	82 ± 9	8.9 ± 0.3	84 ± 3
12	16.8 ± 0.5	16.9 ± 2.8	101 ± 17	17.2 ± 2.7	102 ± 16
13	10.8 ± 0.3	12.3 ± 2.3	114 ± 21	11.3 ± 0.1	105 ± 1
14	11.9 ± 0.4	12.5 ± 1.5	105 ± 13	11.1 ± 0.7	93 ± 6
15	9.7 ± 0.3	11.5 ± 0.3	119 ± 3	11.7 ± 1.5	121 ± 15

Table S2. A comparison of assay parameters for particle-linked assays using SP ICP MS or LA ICP MS as the readout.

Assay type	Detection type	Analyte	LOD	Reference
Homogeneous	Nebulizer SP ICP MS	CEA	0.21 ng/mL	10
Homogeneous	Nebulizer SP ICP MS	CA-125,<br>CEA,<br>CA-19-9	0.43 U/mL,<br>0.23 ng/mL,<br>0.24 U/mL	11
Sandwich heterogeneous in MTP	Nebulizer SP ICP MS	α-fetoprotein	160 ng/mL	12
Sandwich heterogeneous in MTP	Nebulizer SP ICP MS	IgG	0.1 ng/mL	13
Sandwich heterogeneous on MP	Nebulizer SP ICP MS	CEA,<br>CA724,<br>CA19-9	0.1 ng/mL,<br>0.20 U/mL,<br>0.21 U/mL	14
Sandwich heterogeneous on MP	Nebulizer SP ICP MS	CYFRA-21,<br>CEA,<br>CA15-3	0.02 ng/mL,<br>6 pg/mL,<br>0.25 mU/mL	15
Sandwich heterogeneous on MP	Nebulizer SP ICP MS	CEA	6 pg/mL	16
Sandwich heterogeneous on MP	Nebulizer SP ICP MS	MPO, OPN	4 pg/mL,<br>5 pg/mL	17

Direct heterogeneous on PVDF	Analog LA ICP MS	mouse IgG	22 ng/mL	[18]
Direct heterogeneous on PVDF	Analog LA ICP MS	p53	2.6 ng/mL	[19]
Sandwich heterogeneous on NC	IR LA SP ICP MS	HSA,<br>PSA	0.1 ng/mL,<br>0.3 pg/mL	This work

3 References

(1) Hlaváček, A.; Farka, Z.; Mickert, M. J.; Kostiv, U.; Brandmeier, J. C.; Horák, D.; Skládal, P.; Foret, F.; Gorris, H. H. Bioconjugates of Photon-Upconversion Nanoparticles for Cancer Biomarker Detection and Imaging. *Nat. Protoc.* **2022**, *17* (4), 1028–1072. https://doi.org/10.1038/s41596-021-00670-7.

(2) Brandmeier, J. C.; Jurga, N.; Grzyb, T.; Hlaváček, A.; Obořilová, R.; Skládal, P.; Farka, Z.; Gorris, H. H. Digital and Analog Detection of SARS-CoV-2 Nucleocapsid Protein via an Upconversion-Linked Immunosorbent Assay. *Anal. Chem.* **2023**, *95* (10), 4753–4759. https://doi.org/10.1021/acs.analchem.2c05670.

(3) Makhneva, E.; Sklenárová, D.; Brandmeier, J. C.; Hlaváček, A.; Gorris, H. H.; Skládal, P.; Farka, Z. Influence of Label and Solid Support on the Performance of Heterogeneous Immunoassays. *Anal. Chem.* **2022**, *94* (47), 16376–16383. https://doi.org/10.1021/acs.analchem.2c03543.

(4) Brandmeier, J. C.; Raiko, K.; Farka, Z.; Peltomaa, R.; Mickert, M. J.; Hlaváček, A.; Skládal, P.; Soukka, T.; Gorris, H. H. Effect of Particle Size and Surface Chemistry of Photon-Upconversion Nanoparticles on Analog and Digital Immunoassays for Cardiac Troponin. *Adv. Healthc. Mater.* **2021**, *10* (18), 2100506. https://doi.org/10.1002/adhm.202100506.

(5) Pořízka, P.; Vytisková, K.; Obořilová, R.; Pastucha, M.; Gábriš, I.; Brandmeier, J. C.; Modlitbová, P.; Gorris, H. H.; Novotný, K.; Skládal, P.; Kaiser, J.; Farka, Z. Laser-Induced Breakdown Spectroscopy as a Readout Method for Immunocytochemistry with Upconversion Nanoparticles. *Microchim. Acta* **2021**, *188* (5), 147. https://doi.org/10.1007/s00604-021-04816-y.

(6) Máčala, J.; Makhneva, E.; Hlaváček, A.; Kopecký, M.; Gorris, H. H.; Skládal, P.; Farka, Z. Upconversion Nanoparticle-Based Dot-Blot Immunoassay for Quantitative

Biomarker Detection. *Anal. Chem.* **2024**, *96* (25), 10237–10245. https://doi.org/10.1021/acs.analchem.4c00837.

(7) Rueden, C. T.; Schindelin, J.; Hiner, M. C.; DeZonia, B. E.; Walter, A. E.; Arena, E. T.; Eliceiri, K. W. ImageJ2: ImageJ for the next Generation of Scientific Image Data. *BMC Bioinformatics* **2017**, *18* (1), 529. https://doi.org/10.1186/s12859-017-1934-z.

(8) Hu, Z.; Liu, Y.; Gao, S.; Hu, S.; Dietiker, R.; Günther, D. A Local Aerosol Extraction Strategy for the Determination of the Aerosol Composition in Laser Ablation Inductively Coupled Plasma Mass Spectrometry. *J. Anal. At. Spectrom.* **2008**, *23* (9), 1192. https://doi.org/10.1039/b803934h.

(9) Stiborek, M.; Jindřichová, L.; Meliorisová, S.; Bednařík, A.; Prysiazhnyi, V.; Kroupa, J.; Houška, P.; Adamová, B.; Navrátilová, J.; Kanický, V.; Preisler, J. Infrared Laser Desorption of Intact Nanoparticles for Digital Tissue Imaging. *Anal. Chem.* **2022**, *94* (51), 18114–18120. https://doi.org/10.1021/acs.analchem.2c05216.

(10) Huang, Z.; Wang, C.; Liu, R.; Su, Y.; Lv, Y. Self-Validated Homogeneous Immunoassay by Single Nanoparticle in-Depth Scrutinization. *Anal. Chem.* **2020**, *92* (3), 2876–2881. https://doi.org/10.1021/acs.analchem.9b05596.

(11) Huang, Z.; Li, Z.; Jiang, M.; Liu, R.; Lv, Y. Homogeneous Multiplex Immunoassay for One-Step Pancreatic Cancer Biomarker Evaluation. *Anal. Chem.* **2020**, *92* (24), 16105–16112. https://doi.org/10.1021/acs.analchem.0c03780.

(12) Hu, S.; Liu, R.; Zhang, S.; Huang, Z.; Xing, Z.; Zhang, X. A New Strategy for Highly Sensitive Immunoassay Based on Single-Particle Mode Detection by Inductively Coupled Plasma Mass Spectrometry. *J. Am. Soc. Mass. Spectrom.* **2009**, *20* (6), 1096–1103. https://doi.org/10.1016/j.jasms.2009.02.005.

(13) Liu, R.; Xing, Z.; Lv, Y.; Zhang, S.; Zhang, X. Sensitive Sandwich Immunoassay Based on Single Particle Mode Inductively Coupled Plasma Mass Spectrometry Detection. *Talanta* **2010**, *83* (1), 48–54. https://doi.org/10.1016/j.talanta.2010.08.037.

(14) Huang, Z.; Zhao, X.; Hu, J.; Zhang, C.; Xie, X.; Liu, R.; Lv, Y. Single-Nanoparticle Differential Immunoassay for Multiplexed Gastric Cancer Biomarker Monitoring. *Anal. Chem.* **2022**, *94* (37), 12899–12906. https://doi.org/10.1021/acs.analchem.2c03013.

(15) Cao, Y.; Feng, J.; Tang, L.; Mo, G.; Mo, W.; Deng, B. Detection of Three Tumor Biomarkers in Human Lung Cancer Serum Using Single Particle Inductively Coupled Plasma Mass Spectrometry Combined with Magnetic Immunoassay. *Spectrochim. Acta Part B At. Spectrosc.* **2020**, *166*, 105797. https://doi.org/10.1016/j.sab.2020.105797.

(16) Cao, Y.; Mo, G.; Feng, J.; He, X.; Tang, L.; Yu, C.; Deng, B. Based on ZnSe Quantum Dots Labeling and Single Particle Mode ICP-MS Coupled with Sandwich Magnetic Immunoassay for the Detection of Carcinoembryonic Antigen in Human Serum. *Anal. Chim. Acta* **2018**, *1028*, 22–31. https://doi.org/10.1016/j.aca.2018.04.039.

(17) Cao, Y.; Deng, B. Detection Of Myeloperoxidase And Osteopontin In Human Serum Using Single Particle-ICP-MS With MoS2 And ZnS Quantum Dots. *At. Spectrosc.* **2023**, *44* (02), 65–75. https://doi.org/10.46770/AS.2023.042.

(18) Tvrdoňová, M.; Vlčnovská, M.; Vaníčkova, L. P.; Kanický, V.; Adam, V.; Ascher, L.; Jakubowski, N.; Vaculovičová, M.; Vaculovič, T. Gold Nanoparticles as Labels for Immunochemical Analysis Using Laser Ablation Inductively Coupled Plasma Mass Spectrometry. *Anal. Bioanal. Chem.* **2019**, *411* (3), 559–564. https://doi.org/10.1007/s00216-018-1300-7.

(19) Vlčnovská, M.; Štossová, A.; Kuchyňka, M.; Dillingerová, V.; Polanská, H.; Masařík, M.; Hrstka, R.; Adam, V.; Kanický, V.; Vaculovič, T.; Vaculovičová, M. Comparison of Metal Nanoparticles (Au, Ag, Eu, Cd) Used for Immunoanalysis Using LA-ICP-MS Detection. *Molecules* **2021**, *26* (3), 630. https://doi.org/10.3390/molecules26030630.
